# The Orphan Response Regulator Rv3143 Modulates the Activity of the NADH Dehydrogenase Complex (Nuo) in *Mycobacterium tuberculosis via* Protein–Protein Interactions

**DOI:** 10.3389/fcimb.2022.909507

**Published:** 2022-06-28

**Authors:** Renata Płocińska, Karolina Wasik, Przemysław Płociński, Ewelina Lechowicz, Magdalena Antczak, Ewelina Błaszczyk, Bożena Dziadek, Marcin Słomka, Anna Rumijowska-Galewicz, Jarosław Dziadek

**Affiliations:** ^1^ Department of Genetics and Physiology of Mycobacteria, Institute of Medical Biology of the Polish Academy of Sciences, Łódź, Poland; ^2^ Department of Immunology and Infectious Biology, Faculty of Biology and Environmental Protection, University of Łódz, Łódź, Poland; ^3^ Department of Molecular Microbiology, Faculty of Biology and Environmental Protection, University of Łódz, Łódź, Poland; ^4^ Biobank Lab, Department of Molecular Biophysics, Faculty of Biology and Environmental Protection, University of Łódź, Łódź, Poland

**Keywords:** tuberculosis, oxidative respiration, orphan two-component regulators, signal transduction, respiratory chain, NADH dehydrogenase

## Abstract

Two-component signal transduction systems enable mycobacterial cells to quickly adapt and adequately respond to adverse environmental conditions encountered at various stages of host infection. We attempted to determine the role of the Rv3143 “orphan” response regulator in the physiology of *Mycobacterium tuberculosis* and its orthologue Msmeg_2064 in *Mycobacterium smegmatis*. We identified the Rv3143 protein as an interaction partner for NuoD, a member of the type I NADH dehydrogenase complex involved in oxidative phosphorylation. The mutants Δ*rv3143* and Δ*msmeg_2064* were engineered in *M. tuberculosis* and *M. smegmatis* cells, respectively. The Δ*msmeg_2064* strain exhibited a significant reduction in growth and viability in the presence of reactive nitrogen species. The Rv3143-deficient strain was sensitive to valinomycin, which is known to reduce the electrochemical potential of the cell and overexpressed genes required for nitrate respiration. An increased level of reduction of the 2,3,5-triphenyltetrazolium chloride (TTC) electron acceptor in Δ*rv3143* and Δ*msmeg_2064* cells was also evident. The silencing of *ndh* expression using CRISPRi/dCas9 affected cell survival under limited oxygen conditions. Oxygen consumption during entry to hypoxia was most severely affected in the double-mutant Δ*msmeg_2064 ndh^CRISPRi/dCas9^*. We propose that the regulatory protein Rv3143 is a component of the Nuo complex and modulates its activity.

## Introduction

The success of *Mycobacterium tuberculosis* as a human pathogen is based on its ability to adapt to dynamic changes in intracellular and extracellular environments during the infection process. The fundamental feature of this adaptation is the ability to respire and generate energy, at both the replicative and non-replicative stages. To effectively respond to changing environmental conditions, mycobacteria exploit two-component signal transduction systems (TCSSs). A typical TCSS is composed of a membrane-bound histidine sensor kinase that upon detecting environmental signal undergoes autophosphorylation and can transfer the phosphoryl group onto the regulatory domain of the cytosolic response regulator. *M. tuberculosis* possesses 12 such two-component systems, e.g., SenX3/RegX3, PhoP/PhoR, DosR/DosS, MtrA/MtrB, and PdtaS/PdtaR (Zahrt and Deretic, 2000; Morth et al., 2005; Dadura et al., 2017). The genome of *M. tuberculosis* possesses information for six response regulators (Rv0195, Rv0260c, Rv0818, PdtaR, Rv2884, and Rv3143). Antczak and colleagues reported the role of NnaR (Msmeg_0432) as a regulator of nitrogen metabolism in *Mycobacterium smegmatis*  (Antczak et al., 2018). *rv3143* gene was found to be upregulated in DosR mutant, in *M. tuberculosis*  (Kendall et al., 2004). Recently, it was shown that Rv3143 increases antibiotic sensitivity by regulating cell wall permeability in *M. smegmatis*  (Dong et al., 2020). The role of orphaned elements in bacteria is very fragmentary and remains to be determined. In microorganisms such as mycobacteria, similar to human mitochondria, membrane-bound ATPase catalyzes the synthesis of ATP when an electrochemical gradient (proton motive force (PMF)) is imposed across the cell membrane (Maloney et al., 1974). PMF is generated by an electron transport chain that acts as a proton pump across the membrane during respiration. The respiratory chain in *M. tuberculosis* is composed of nine respiratory dehydrogenases and four terminal oxidoreductases (for review, see (Cook et al., 2014). Since PMF is essential for the viability of replicative and dormant *M. tuberculosis*, the respiratory chain is considered a promising drug target for new anti-tuberculosis drug development. Under aerobic conditions, the major respiratory terminal oxidoreductase in mycobacteria is the *bc_1_-aa_3_* cytochrome *c* supercomplex (Matsoso et al., 2005; Megehee et al., 2006). Menaquinol-cytochrome *c* oxidoreductase (*bc_1_*), encoded by *qcr*CAB, and *aa_3_*-cytochrome *c* oxidase, encoded by *cta*BCDE, belong to the heme-copper respiratory oxidase family (Boshoff and Barry, 2005; Matsoso et al., 2005; Megehee et al., 2006). The inactivation of the *bc_1_-aa_3_* complex in *Mycobacterium* (*Mycolicibacterium*) *smegmatis* leads to the upregulation of cytochrome *bd*-type menaquinol oxidase, encoded by *cydABDC*, which is also present in other species of the *Mycobacterium* genus (Matsoso et al., 2005). A *cydC* mutant of *M. tuberculosis* was attenuated under transition from acute to chronic infection in mice, and CydC is involved in the persistence of *M. tuberculosis* in isoniazid-treated mice (Shi et al., 2005; Dhar and McKinney, 2010). Imidazopyridine amide, Q203, targeting the respiratory cytochrome bc1 complex, was reported to be efficacious in a mouse model of tuberculosis at a dose lower than 1 mg per kg of body weight (Pethe et al., 2013). More recently, despite the affinity of Q203 for the *bc_1_-aa_3_* complex, the drug was shown to be only bacteriostatic and is not able to affect drug-tolerant persisters (Kalia et al., 2017). However, Q203 presented bactericidal activity against an *M. tuberculosis* mutant carrying inactivated *cyd*AB genes encoding cytochrome *bd* oxidase (Kalia et al., 2017). Furthermore, the downregulation or inactivation of *ctaE-qcrCAB*, *ctaC*, and *ctaD* reduced but did not prevent the growth of mutants. The Δ*ctaE-qcrCAB M. tuberculosis* mutant was attenuated in the acute phase of mouse infection, but by day 28 post-infection, the strain had reached the same titer as control wild-type *M. tuberculosis* and showed no persistent defect thereafter (Beites et al., 2019). The upregulation of *cydA* in the *bc_1_-aa_3_* mutant strain as well as the construction and analysis of mutants defective in the synthesis of both *bc_1_-aa_3_* and *bd* oxidases clearly demonstrated that *M. tuberculosis* requires the *bc_1_-aa_3_* complex to achieve an optimal growth rate; however, *bd* oxidase alone can support *M. tuberculosis* growth and persistence *in vitro* and *in vivo* (Beites et al., 2019). The use of a marmoset (non-primate monkey) tuberculosis infection model confirmed that the efficient inhibition of cytochrome *bc_1_-aa_3_* oxidase allows the reduction of inflammation, but only a subset of bacilli were affected, while the bacilli present in granulomas exacerbate disease by increasing cavitation (Beites et al., 2019).

Tubercle bacilli possess a single copy of proton-pumping type I dehydrogenase (NDH-1, Nuo) and two copies of NADH dehydrogenases type II (NDH-2), encoded by *ndh* and *ndhA* genes, that transfer electrons to the quinone pool *via* a ping-pong reaction mechanism (Yano et al., 2006). Fast-growing *M. smegmatis* possesses only a single copy of NDH-2 (Ndh), but it carries approximately 95% of the total NADH oxidation measured in this model organism (Vilchèze et al., 2005). NADH-oxidizing activity in *M. tuberculosis* is also mediated mainly by NDH-2, with NDH-1 activity lower than 50% (Cook et al., 2014). NDH-1 of *M. tuberculosis* is encoded by a *nuo* operon consisting of 14 subunits (*nuoA-N*). This operon, except for the pseudogene *nuoN*, is missing in *Mycobacterium leprae* (Cole et al., 2001). NuoB–G are peripheral membrane proteins located on the cytoplasmic side, while NuoA, H, and J–N are located in the membrane section of the complex (Cook et al., 2014; Schut et al., 2016). The *nuo* operon in *M. tuberculosis* was reported to be essential for neither growth nor persistence under oxygen depletion conditions in a Wayne model (Sassetti et al., 2003; Rao et al., 2008; Griffin et al., 2011; DeJesus et al., 2017). The construction and analysis of various *M. tuberculosis* mutants defective in the synthesis of one or more NADH dehydrogenases (Ndh, NdhA, and/or Nuo) showed that Ndh is the main NADH dehydrogenase in tubercle bacilli (Vilchèze et al., 2018). The authors were able to inactivate *ndhA*, *nuoAN*, and both dehydrogenases together with no serious phenotype determined. In contrast, mutants defective in the synthesis of Ndh or both Ndh and NuoAN presented several growth defects *in vitro* as well as *in vivo*, with the Δ*ndh*Δ*nuoAN* double mutant most severely attenuated in mice (Vilchèze et al., 2018). The authors were not able to inactivate both NDH-2 dehydrogenases in *M. tuberculosis*, concluding that at least one NDH-2 dehydrogenase might be essential for the viability of mycobacteria (Vilchèze et al., 2018). Since NDH-2 seems to be the major NADH dehydrogenase in *M. tuberculosis*, it is considered an attractive target for new drug development (Weinstein et al., 2005; Shirude et al., 2012; Harbut et al., 2018; Murugesan et al., 2018). More recently, the essentiality of NDH-2 was shown to be conditional and dependent on the presence of fatty acids. The *M. tuberculosis* mutant Δ*ndh-2* appeared to be attenuated in the acute phase of infection, but its persistence was not significantly affected (Beites et al., 2019). Since NDH-2 is not required for *M. tuberculosis* in media containing short-chain fatty acids or cholesterol, the treatment of tuberculosis by targeting NADH dehydrogenase might require efficient inactivation of all three enzymes, Ndh, NdhA, and the Nuo complex.

Here, we applied a suite of microbiology, molecular biology, and biochemistry methods to identify the role of an “orphan” regulatory protein of the two-component system family, Rv3143, which we found to be a component of the NDH-1 dehydrogenase complex. The inactivation of *rv3143*, as well as its ortholog *msmeg_2064* in *M. smegmatis*, especially in the context of *ndh* depletion, affects the functionality of the respiratory chain in mycobacteria.

## Materials and Methods

### Bacterial Strains and Growth Conditions

The *Escherichia coli* strains used in this study were cultured in Luria–Bertani (LB) broth or on agar plates supplemented with ampicillin (50 μg/ml), kanamycin (50 μg/ml), hygromycin (200 μg/ml), and chloramphenicol (34 μg/ml) (Sigma-Aldrich, Missouri, USA). The *M. tuberculosis* strains were grown in 7H9 Middlebrook liquid media supplemented with 10% OADC enrichment (oleic acid, albumin, dextrose, and catalase) and Tween-80 (0.05%) or 7H10/OADC agar plates (Difco, Baltimore, USA). The *M. smegmatis* strains were propagated in Middlebrook 7H10 agar or 7H9 media with OADC and Tween-80 except for experiments under hypoxia where AD enrichment was used. The following antibiotics were used to culture the *M. tuberculosis* and *M. smegmatis* strains: kanamycin (25 μg/ml) and hygromycin (50 μg/ml). To induce *ndh* depletion in *M. smegmatis* strains, anhydrotetracycline (aTc; 100 ng/ml; Sigma-Aldrich, Missouri, USA) was added. All strains used in this study are listed in **Supplementary Table S4**.

### Gene Cloning Strategies

All procedures associated with gene cloning into vectors (plasmid isolation, ligation, and transformation) were performed according to the protocols by Sambrook and Russell 2001 (Joseph Sambrook, 2001). All PCR products were generated using KAPA HiFi DNA Polymerase (KAPA Biosystems, Wilmington, MA, USA) and directly cloned into the linearized vector pJET 1.2/blunt (Thermo Fisher Scientific, Waltham, MA, USA). The genes of interest were sequenced, released using restriction endonucleases, and cloned into final vectors. The plasmids and primers used in this study are listed in **Supplementary Table S4**.

### Construction of Gene Replacement Vectors and Complementation Plasmids

Mutant strains lacking functional Rv3143 and MSMEG_2064 proteins were constructed according to the homologous recombination protocol by Parish and Stocker (Parish and Stoker, 2000). First, non-functional *msmeg_2064* and *rv3143* genes containing an internal deletion region and marker cassette enabling easy selection of recombinants were cloned into a suicidal p2Nil vector. The 5′ fragments of *msmeg_2064* and *rv3143* (35 and 85 bp, respectively) genes with upstream regions (1,020 and 1,268 bp, respectively) were cloned into the suicidal recombination vector p2Nil. Next, the 3′ fragments of genes of interest (224 and 193 bp) with downstream regions (1,380 and 1,472 bp) along with the PacI screening cassette carrying *lacZ* and *sacB* genes from the pGOAL17 vector were cloned as described previously (Dadura et al., 2017; Antczak et al., 2018). The final plasmids pKW5 and pKW10 were used for a two-step mutant selection protocol. The complementation plasmids carrying native *msmeg_2064* and *rv3143* genes along with their putative promoter sequences were cloned into the pKW08Lx and pMV306 vectors and transformed into the appropriate mutant cells.

### Disruption of the *Mycobacterium tuberculosis rv3143* and *Mycobacterium smegmatis msmeg_2064* Genes at Their Native Chromosomal Loci

The final gene replacement vectors pKW5 carrying an internal deletion in *msmeg_2064* and pKW10 (deletion in *rv3143*) were treated with 0.2 mM of NaOH and electroporated into *M. smegmatis* and *M. tuberculosis* cytoplasm. The obtained blue colonies sensitive to sucrose and Kan^R^ were single crossover (SCO) recombinants possessing the wild-type and mutant copies of the studied genes. The SCO strains were further processed for selection of double crossover (DCO) mutants that were white, resistant to sucrose, Kan^S^, and retaining only one copy of the wild-type or mutated gene. The genotypes of the obtained DCO mutants Δ*msmeg_2064* and Δ*rv3143* were confirmed by Southern blotting hybridization using probes homologous to the investigated genes and the Amersham ECL Direct Nucleic Acid Labelling System (Amersham Pharmacia Biotech UK Ltd., Buckinghamshire, UK).

### Growth Kinetics and Survival Analyses in the Presence of Reactive Oxygen and Nitrogen

The *M. smegmatis* strains grown in 7H9/AD medium up to the logarithmic stage were diluted to OD_600_ 0.1 and further propagated at 37°C in addition to reactive nitrogen forms–DETA NONOate and reactive oxygen species–menadione at final concentrations of 1,000 and 150 μM, respectively. The absorbance at 600 nm was measured every 3 h throughout the duration of the experiment. To determine the number of viable cells (colony-forming units (CFU) per ml) after 6, 9, 12, and 24 h of growth, bacteria were diluted and spread on 7H10 agar plates. The colonies obtained were counted after 3–5 days of incubation at 37°C, and an Excel file was used for calculations. The *M. tuberculosis* strains were cultured in the same media with the addition of DETA NONOate (25 μM) and menadione (10 μM). As we reported earlier, the growth of *M. tuberculosis* wild-type cells, propagated in the presence of 25 and 50 µM of DETA NONOate, was inhibited by approximately 30% and 50%, respectively. The growth of *M. tuberculosis* was reduced by about 50% and 10% in the presence of menadione in the concentration of 40 and 10 µM, respectively (Brzostek et al., 2014). In the case of the *M. smegmatis* wild-type strain, 1,000 µM of DETA NONOate was required to achieve a growth reduction of about 50%. Bacterial growth was monitored by measuring the turbidity (OD_600_) of each culture for 11 days of the experiment. Mycobacterial viable counts were determined as CFU per ml on days 4 and 9 of incubation, as described above.

### Minimum Inhibitory Concentration

The microplate Alamar blue assay (MABA test) was applied to define the minimum inhibitory concentration (MIC) value (the lowest concentration of compound that prevents the growth of microorganisms) as described by Franzblau et al. (1998) and Dadura et al. (2017). The *M. tuberculosis* wild-type and Δ*rv3143* strains were propagated in a 7H9/OADC medium supplemented with casein hydrolysate (0.1%) and Tween-80 (0.05%) up to the logarithmic phase. Next, the cells were diluted to a 1.0 McFarland turbidity and 10-fold further in the same media. Thus, the prepared bacterial suspension (100 μl) was transferred to 96-well flat-bottom plates containing 100 μl of 7H9 medium with 2-fold dilutions of the concentration ranges of the tested compounds: valinomycin (1.5–0.125 μg/ml), CCCP-carbonyl cyanide *m*-chlorophenyl hydrazine (10-0.312 ug/ml), and trifluoperazine (28-0.437 ug/ml) (Sigma-Aldrich, St. Louis, MO, USA). The microtiter plate was incubated at 37°C for 7 days, and 25 μl of Alamar blue solution (Thermo Fisher Scientific, Waltham, MA, USA) was added to each well and incubated for an additional 48 h. The susceptibility of the tested strains was assessed based on the change in color from blue to pink, based on visual inspection. Wells containing only bacteria, medium, or compound were used as controls in this experiment, and the MABA test was repeated independently three times.

### Construction of CRISPR-Cas Strains

The CRISPR-Cas system was applied to lower the expression level of *ndh* gene in the presence of the inducer in the Δ*msmeg_2064* mutant and *M. smegmatis* wild-type strain (Rock et al., 2017; Korycka-Machała et al., 2020). For this purpose, a 20-nucleotide DNA sequence complementary to the *M. smegmatis ndh* gene (*msmeg3621*) and 2 nucleotides away from the PAM sequence (5′-AGAAG-3′) was synthesized and cloned into the pLJR962 vector linearized with BsmBI endonuclease. The obtained pKW16 plasmid (**Supplementary Table S4**) was electroporated into *M. smegmatis* wild-type and Δ*msmeg_2064* mutant competent cells. Sequences of primers used to silence *ndh* gene are listed in **Supplementary Table S4**.

### Survival Assessment of CRISPR-Cas Strains Under Hypoxia and Discoloration of Methylene Blue

The survival of mycobacterial strains was determined by growth kinetics and the viable CFU/ml. All studied strains were grown in 7H9 media supplemented with 10% AD and 0.05% Tween-80 up to OD_600_ 0.6–0.8 at 37°C. To silence *ndh* gene, the strains were propagated in the presence of aTc (100 ng/ml) for 16 h. Next, to obtain hypoxic conditions, cells were diluted to an OD_600_ of 0.3, and 12 ml of each culture was transferred to a 15-ml Falcon tube, tightly closed, sealed with parafilm, and incubated at 37°C for 6 h, 24 h, and 10 days with shaking. The different Falcon tubes have been prepared for each time point, in order not to disturb the hypoxic condition. To the control tube, the oxygen consumption indicator methylene blue was added at a final concentration of 6 μg/ml. The kinetics of growth was determined by measuring the optical density at 600 nm at 6 h, 24 h, and 10 days. Serial dilutions of the cells at the same time points were plated on 7H10 agar plates, colonies were counted, and CFU/ml was calculated. The discoloration of methylene blue was determined by measuring the absorbance at 600 nm every hour until the complete discoloration of the indicator. The experiment was repeated three times. Hypoxic conditions and *ndh* depletion were performed in the same way as described above.

### Cloning, Expression, and Purification of Rv3143 and NuoD Proteins

The sequence coding the Rv3143 was cloned into pGEX-6P-2 (Addgene, Watertown, MA, USA), enabling purification of the tested protein fused to a glutathione S-transferase (GST) tag, and the sequence coding NuoD protein was cloned into pE-SUMO (LifeSensors Inc., Malvern, PA, USA) expression vector fused to a His tag. The overexpression of Rv3143-GST and pE-SUMO-NuoD was carried out in the *E. coli* BL21 (DE3) strain in LB media at 37°C with shaking until the OD_600_ reached 0.6–0.8. Next, the cells were cooled to 20°C, the expression was induced with isopropyl β-d-1-thiogalactopyranoside (IPTG) measuring 0.4 mM (Rv3143-GST) and 0.2 mM (pE-SUMO-NuoD), and the cells were cultured overnight at 20°C. Purification of recombinant proteins was performed from cell pellets obtained from 1 L of induced culture using affinity chromatography. The cell pellet was suspended in phosphate-buffered saline (PBS) buffer, sonicated using an ultrasonic probe (10 × 10 s), and centrifuged. Rv3143-GST was purified using agarose resin with immobilized glutathione, GST GraviTrap™ columns (GE Healthcare, Chicago, IL, USA), and elution buffer (50 mM of Tris-HCl, pH 8.0, 10 mM of reduced glutathione). Thirty milliliters of washing buffer (50 mM of Tris-HCl, pH 8.0, 10% glycerol) was used to remove reduced glutathione, and chosen fractions of purified Rv3143-GST protein were concentrated on a Vivaspin® 6, 10 kDa MWCO concentrator (GE Healthcare, IL, USA). The harvested cells of pESUMO-NuoD were suspended in binding buffer, pH 7.5 (50 mM of Tris-HCl, 500 mM of NaCl, and 0.1% sodium dodecyl sulfate (SDS)) with 100 μg/ml of lisozyme, disintegrated by sonication, and precleared by centrifugation, and supernatant with 2 M of hexylene glycol was incubated in an ice bath for 1.5 h with shaking. The recombinant protein was purified by applying His pure Ni-NTA resin (Thermo Fisher Scientific, Waltham, MA, USA) and elution buffer containing 500 mM of imidazole. To obtain a purer protein preparation, a HiTrap SP FF column with an AKTA start (GE Healthcare, IL, USA) system was applied, and the pSUMO-NuoD protein was eluted in a NaCl gradient with buffers containing 50 mM of Tris-HCl and 10% glycerol. To remove imidazole, the protein was passed through a Sephadex G-25 column and concentrated on a Vivaspin® 6, 10 kDa MWCO concentrator.

### Preparation of Mouse Polyclonal Anti-Rv3143 Antibodies

The Rv3143 recombinant protein was concentrated on a Novagen concentrator (Merck KGaA, Darmstadt, Germany) to a final concentration of 1 mg/ml and then used for immunization of 8- to 12-week-old female mice of the BALB/c strain. The recombinant protein was emulsified with incomplete Freund’s adjuvant (IFA) (Sigma-Aldrich, Missouri, USA) at a ratio of 1:1 (v/v), and the protein/adjuvant mixture was administered subcutaneously using three doses at 2-week intervals. The final amounts of mycobacterial recombinant antigen used for immunization were 100 µg (1st dose) and 80 µg (2nd and 3rd doses). Two weeks after the last booster injection, mice were anesthetized with 40 mg per kilogram of body weight sodium pentobarbital, blood samples were collected from the orbital sinus, and laboratory animals were further euthanized using barbiturate overdose. Mouse sera were prepared from the collected blood samples, and both the titers and optimal working dilutions of polyclonal anti-Rv3143 IgG immunoglobulins (primary antibodies) were determined using indirect ELISA with recombinant Rv3143 protein as a coating antigen (20 µg/ml) and horseradish peroxidase (HRP)-labeled goat polyclonal antimouse IgG immunoglobulins as secondary antibodies (Jackson ImmunoResearch, West Grove, PA, USA) diluted 1:2,000. The resulting immune complexes were detected using a mixture of ABTS chromogen (2,2′-azino-bis(3-ethylbenzothiazoline-6-sulfonic) acid at a concentration of 1 mg/ml (Sigma-Aldrich, St. Louis, MO, USA) and H_2_O_2_ (Sigma-Aldrich, St. Louis, MO, USA) as an HRP substrate in phosphate-citrate buffer, pH 4.5. The optimal working dilution of secondary antibodies was established in the preliminary titration experiments.

The laboratory BALB/c mice used for immunization were raised under standard conventional conditions approved by the Polish Ministry of Science and Higher Education Animal Facility of the Institute Microbiology, Biotechnology and Immunology, Faculty of Biology and Environmental Protection, University of Lodz. The applied experimental procedures were approved and conducted according to guidelines provided by the appropriate Polish Local Ethics Commission for Experiments on Animals No. 9 in Lodz (Agreement 54/ŁD1/2011).

### Protein Complex Purification

To identify the protein complexes for Rv3143, the integration pKW08 vector possessing the *rv3143* sequence fused with enhanced green fluorescent protein (eGFP) under a tetracycline-induced promoter was constructed. The final plasmid pKW17 was electroporated into *M. tuberculosis-*competent cells. The overexpression of Rv3143-eGFP and purification of protein complexes were performed according to a published protocol (Płociński et al., 2014). Actively growing *M. tuberculosis* cells expressing Rv3143-eGFP in Middlebrook 7H9/OADC media with an optical density OD_600 of_ 0.6 were induced with 50 ng/ml of tetracycline and incubated at 37°C for 48 h. Three hundred milliliters of induced cells was centrifuged at 4,500 rpm for 10 min at 4°C and washed twice with 30 ml of washing buffer (50 mM of HEPES, pH 7.4, 150 mM of NaCl). The cell pellet obtained was suspended in 9 ml of cold lysis buffer, transferred to BIGB-Lysing MATRIX B-tube (MP Biomedicals, Irvine, CA, USA), and homogenized five times for 20 s at 6.0 m/s using Ms disruptor system with Quick prep adapter (MP Biomedicals, Irvine, CA, USA) with a 5-min break between cycles on ice. Next, the clarified supernatant after spinning was incubated with GFP-trap agarose resin (ChromoTek, Planegg-Martinsried, Germany) for 2 h in a cold room with rotation. The column was washed three times with 10 ml of IPP150 buffer. In some experiments, bis(sulfosuccinimidyl)suberate (BS3) was added at a final concentration of 2 mM (Thermo Fisher Scientific, Waltham, MA, USA); a crosslinker was added according to the manufacturer’s instructions (Thermo Fisher Scientific, Waltham, MA, USA), incubated for 45 min at room temperature in the dark and washed twice with 10 ml of tobacco etch virus (TEV) buffer. Next, 3 μl of TEV protease (Promega, Madison, WI, USA) in 400 μl of TEV buffer was incubated with the column for 4 h at room temperature followed by overnight incubation at 4°C. To precipitate the protein complexes obtained, pyrogallol red–molybdate (PRM) buffer was added and incubated for 5 h at room temperature followed by overnight incubation at 4°C. Samples were centrifuged for 25 min at 12,000 rpm at 25°C, and the resulting pellet was subjected to high-performance liquid chromatography coupled to tandem mass spectrometry (LC-MS/MS) analysis using Orbitrap Velos as a service at the Institute of Biochemistry and Biophysics Polish Academy of Sciences in Warsaw. The procedure is described in detail elsewhere (Płociński et al., 2014). The raw MS data were analyzed with MaxQuant 1.6.17.0 using default settings and label-free analysis against an *M. tuberculosis* proteome (mycobrowser v.3 *M. tuberculosis* database). Raw data for samples treated with BS3 crosslinker were converted to mzML format with the MSConvert application of the ProteoWizard, and protein–protein crosslinks were identified using Kojak version 1.5.3.

### Phylogenetic Analysis of NuoD Proteins

NuoD phylogenetic analysis was performed with an online ETE3 pipeline provided by the database of the Kyoto Encyclopedia of Genes and Genomes (KEGG). First, the *M. tuberculosis* NuoD gene cluster analysis function was applied to pull out all the bacterial NuoD proteins that were most relevant to the downstream analyses. The threshold was set to 100, and the gap size was set to 0. NuoD alignment and phylogenetic reconstructions were performed using the function “build” of ETE3 v3.1.1 (Huerta-Cepas et al., 2016) as implemented on GenomeNet (https://www.genome.jp/tools/ete/). Alignment was performed with MAFFT v6.861b with the default options (Katoh and Standley, 2013). The tree was constructed using FastTree v2.1.8 with default parameters (Price et al., 2009). Values at nodes are SH-like local support. The ETE3 Python programming package, with necessary dependencies, was used for data visualization. The co-occurrence of NuoD variants with Rv3143 orthologs was calculated from the gene cluster information and visualized on the phylogenetic tree.

### Pull-Down Assay

A pulldown assay was performed to investigate the interaction between Rv3143 and NuoD proteins. A 200-μl solution of Rv3143-GST (50 µg) suspended in binding buffer (50 mM of Tris-HCl, pH 8.0, 150 mM of NaCl, 0.5% TitonX, and 10% glycerol) was mixed with GST magnetic beads and incubated for 1 h in an ice bath with swinging. The non-bound fraction was removed using a magnetic separator, and beads were washed six times with 1 ml of washing buffer (50 mM of Tris-HCl, pH 8.0, 150 mM of NaCl, 0.5% TitonX, 20 mM of imidazole, and 10% glycerol). Next, NuoD-His (50 µg) in binding buffer was incubated with magnetic beads for 1 h and washed six times, and protein complexes were eluted with 90 μl of buffer containing 0.3 M of imidazole. Incubation of proteins with magnetic beads to which they did not show affinity was the control in this experiment. Protein fractions were resolved on a 12% SDS–polyacrylamide gel electrophoresis (PAGE) gel and transferred to polyvinylidene difluoride (PVDF) membranes, and protein complexes were immunodetected with rabbit anti-GST (Sigma, St. Louis, MO, USA) and anti-His (Sigma, St. Louis, MO, USA) antibodies, washed with TBS-T (6 × 5 min), and probed with anti-rabbit secondary antibodies for 1.5 h. Immunoblots were processed with enhanced chemiluminescence (ECL) solution as a substrate for HRP following the manufacturer’s instructions (Western Sun Luinol-Enhancer Solution, Cyanagen, Bologna, Italy), exposed to X-ray film, and scanned using a Medical X-ray Processor (Kodak, Rochester, NY, USA).

### Analysis of Respiratory Efficiency of Mutant Cells by Reduction of 2,3,5-Triphenyltetrazolium Chloride

The Δ*rv3143* and Δ*msmeg_2064* cells from the logarithmic phase of growth were diluted in 7H9/OADC media to 1 × 10^6^ cells/ml and transferred to a microtiter 96-well plate (200 μl). Sterile 2,3,5-triphenyltetrazolium chloride (TTC) solution at a final concentration of 0.625 mg/ml was added, and the plate was incubated at 37°C for 0, 2, and 6 h. The Benchmark Plus Microplate Spectrophotometer (Bio-Rad Laboratories, Hercules, CA, USA) was used to measure the amount of red formazan at a 480-nm wavelength. The significant differences between the studied strains were estimated using Student’s *t*-test.

### RNA Isolation, Quantitative Real-Time PCR, and Total RNA Sequencing

*M. smegmatis* cells were cultured in Middlebrook 7H9 liquid medium supplemented with AD for 16 h at 37°C with or without aTc. When the OD_600_ reached 0.6, 50 ml of culture was centrifuged at 4,500 rpm at 4°C for 20 min, and the cell pellet was stored at −70°C. Total RNA was extracted using TRIzol LS reagent (Thermo Fisher Scientific, Waltham, MA, USA), 0.1-mm silica spheres, and an MP disruptor system (MP Biomedicals, Irvine, CA, USA) as previously described (Pawelczyk et al., 2011; Dadura et al., 2017; Antczak et al., 2018). A Turbo DNA-*free*™ Kit (Ambion Inc., Austin, TX, USA) was used to remove DNA contamination following the manufacturer’s instructions, and RNA quantity was assessed using a NanoDrop 2000 spectrophotometer (MP Biomedicals, Irvine, CA, USA). Reverse transcription was performed using SuperScript III First-Strand Synthesis Super Mix (MP Biomedicals, Irvine, CA, USA) and random hexamers. The expression profile of the studied genes was analyzed by qRT-PCR using Maxima SYBR green qPCR master mix (MP Biomedicals, Irvine, CA, USA) and a 7900HT real-time PCR system (Applied Biosystems, Foster City, CA, USA) as described previously (Pawelczyk et al., 2011; Dadura et al., 2017; Antczak et al., 2018). The total reaction of 25 μl containing 1× Maxima SYBR green qPCR master mix, 50 ng of cDNA, and 0.3 μM of each primer was activated at 95°C for 10 min. Next, 40 cycles of denaturation at 95°C for 20 s were followed by annealing at 60°C for 30 s and extension at 72°C for 30 s. After qRT-PCR, the melting curve was determined to verify that a single specific product was generated. The relative fold changes in gene expression were calculated using the double-delta method (2^−ΔΔCT^). The number of tested transcripts was normalized to the reference gene *msmeg_2758* (*sigA*) and then compared to the control strain.

The total RNA sequencing libraries were prepared as described in detail in our previous work (Płociński et al., 2019). Briefly, 2 µg of DNAse Turbo-treated, AMPure XP bead purified RNA was ribodepleted with a RiboZero Bacteria kit (Illumina, San Diego, CA, USA). Sequencing libraries were generated with a KAPA-stranded RNA-Seq kit according to the manufacturer’s protocol (Roche Diagnostics, Rotkreuz, Switzerland). The resulting adapter-ligated, PCR-amplified cDNA libraries were subjected to sequencing on a NextSeq500 sequencer using the NextSeq500/550 v.2 sequencing kit (Illumina, San Diego, CA, USA), and approximately 5 to 10 million paired-end reads were sequenced for each sample. The sequencing data were processed using a suite of bioinformatic scripts and programs and mapped to the H37Rv reference genome as previously published (Płociński et al., 2019). Differential gene expression analysis was performed with the Degust RNA-Seq analysis platform using default parameters (http://degust.erc.monash.edu/, originally designed by D. R. Powell). The numerical data obtained from transcriptomic analysis were imported into Python Pandas data frame format, and the Seaborn package was used to draw the results as figure elements (Płociński et al., 2019).

## Results

### Rv3143 Protein Interacts With NADH Dehydrogenase Type I Complex Proteins

**Figure 1:**
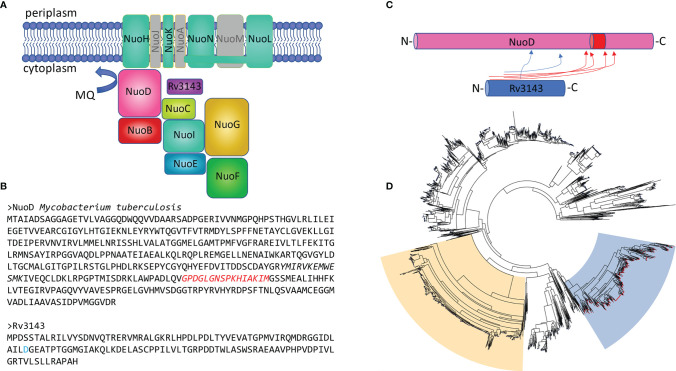
The orphan response regulator Rv3143 is a component of the NuoA–N complex in actinobacteria. **(A)** Graphical summary of the interaction network of Rv3143 with NADH dehydrogenase complex I in *Mycobacterium tuberculosis* based on a single-step affinity purification method coupled with mass spectrometry. Nuo components that were not copurified with eGFP-Rv3143 used as bait were grayed out (the figure layout was modified from Schut et al. [(Schut et al., 2016)]. **(B)** Sequences of *M. tuberculosis* NuoD and Rv3143 are shown. Putative, conserved phosphorylation site (D, aspartic acid) is marked in blue on the Rv3143 sequence. The amino acid insertion sequence (marked in red) found in actinobacterial NuoD proteins may be involved in mediating the interaction between NuoD and Rv3143, as indicated by mass spectrometry BS3 crosslinking analysis performed with Kojak software. Crosslinks with Kojak scores higher than 0.5 are shown in the rudimentary cartoon figure **(C)**. Insertion of the amino acid stretch depicted in panel B is common in actinobacteria, and its presence correlates with the co-occurrence of Rv3143 orthologs in these bacteria. The phylogenetic tree is shown **(D)**, where the blue background indicates the NuoD cluster for which genomic co-occurrence of Rv3143 orthologs is evident (red nodes on each tree leaf). The phylogenetic cluster for NuoCD and NuoBCD chimeric proteins, common in proteobacteria, is indicated with moccasin color for comparison.

Rv3143 is annotated as a putative “orphan” response regulator of a two-component system, and the protein contains no DNA-binding domain, which indicates that it does not operate as a transcription factor. To identify the function of Rv3143, we employed our previously published method to screen for potential partners of the protein investigated in *M. tuberculosis*. The gene fusion encoding Rv3143 tagged to *eGFP-tag* (eGFP) was constructed, cloned into the integration pKW08 vector under control of the *P_tet_* promoter, and then introduced into *M. tuberculosis*. The recombinant strain was cultured to the logarithmic phase of growth (OD_600_ at 0.6) when the expression of the bait protein was induced with tetracycline. Bacterial cells were allowed to overproduce GFP-Rv3143 protein and were collected and lysed, and the released proteins were purified on anti-GFP agarose resin, as described in the *Materials and Methods*. GFP-Rv3143 fusion protein and the potential interacting proteins binding to the bait, Rv3143, were recovered from the resin with TEV protease cleavage, and one of the samples was crosslinked with the BS3 crosslinker, which introduces stable amide bonds. The interacting proteins were discovered by MS analysis. The specificity of the purification process was confirmed by Rv3143 being among the top three most abundant proteins identified in the MS samples. Both samples containing the BS3 crosslinker and devoid of the crosslinker were rich in cytoplasmic components of the Nuo protein complex. Among the 30 most abundant proteins copurified with bait, we identified NuoE, NuoG, NuoF, NuoI, NuoD, NuoC, and NuoB (**Figure 1A** and **Supplementary Table S1A**). While much less abundant, we could also identify NuoL, NuoH, NuoN, and NuoK membrane proteins copurified with Rv3143. Mapping the interaction network with the BS3 crosslinker allowed us to identify a possible interaction between Rv3143 and the NuoD protein. Not only did the NuoD and Rv3143 pair have the highest number of crosslink instances identified, but the crosslinks were also assigned the highest score by Kojak software (**Figure 1C** and **Supplementary Table S1B**). Since the orthologs of Rv3143 are often within the same operon as the NuoA–N proteins in actinobacteria (e.g., *M. smegmatis*), we tested the co-occurrence of NuoD and Rv3143 orthologs in other bacteria. Based on a KEGG gene cluster search performed with mycobacterial NuoD, 3,503 orthologs were identified, and their sequences were fetched for phylogenetic analysis with the ETE3 pipeline. Interestingly, NuoD orthologs containing insertions inside the protein sequence clustered separately from other ortholog sequences (insertion marked in **Figure 1B**). The co-occurrence of our putative response regulator was addressed at the same time. The results clearly indicated that the actinobacterial NuoD orthologs, for which an ortholog of Rv3143 co-occurs on the bacterial genome, cluster away from other bacterial species (**Figure 1D**). Of 516 NuoD variants containing the extra stretch of amino acids in their sequence and thus forming a phylogenetic cluster, 513 were linked with co-occurrence of Rv3143 ortholog on their genomes at the same time.

**Figure 2:**
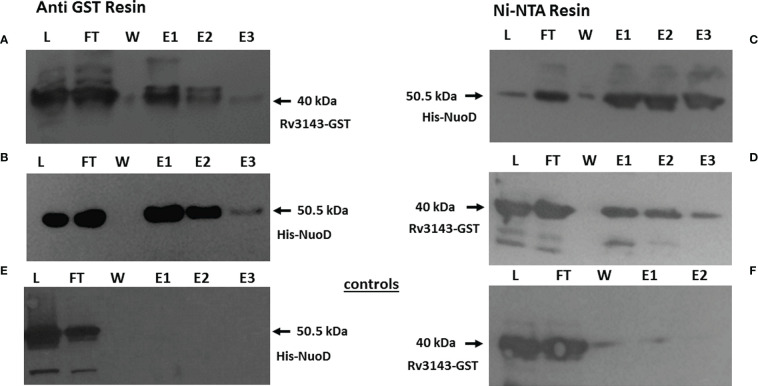
Rv3143 protein interacts with NuoD *in vitro*. Immunoblots of NuoD pulled down with Rv3143 on anti-glutathione S-transferase (anti-GST) resin **(A, B)**. Immunoblots of Rv3143 pulled down with NuoD on Ni-NTA resin **(C, D)**. Bound proteins were eluted with imidazole, resolved on sodium dodecyl sulfate–polyacrylamide gel electrophoresis (SDS-PAGE) gels, transferred to polyvinylidene difluoride (PVDF) membranes, and probed with α-His and α-GST antibodies. L, load; FT, flow-through; W, wash; E, elution. **(E, F)** Non-specific interactions of the tested proteins.

To verify the protein–protein interaction identified by MS, we purified recombinant versions of His-NuoD and GST-Rv3143 as full-length polypeptides. NuoD was purified as a fusion protein with the N-terminal polyhistidine (6-His) tag, and Rv3143 was tagged with a GST tag. GST-Rv3143 and His-NuoD were incubated with anti-GST magnetic beads. We also immobilized His-NuoD protein with Ni-NTA magnetic beads and incubated it with Rv3143 protein. Western blotting analysis with tag-specific antibodies revealed the presence of both tested proteins in eluates obtained from anti-GST and Ni-NTA magnetic beads after extensive washing, confirming the interaction between Rv3143 and NuoD proteins (**Figure 2**). In the control experiments, the potential non-specific interaction of the tested proteins with no-affinity resin was verified, with no detection of the investigated proteins by Western blotting (**Figures 2E, F**). We have also performed the pull down between GST alone and NuoD with no interactions detected. The results are introduced into the Supplementary materials **Figure S2**.

### Rv3143/MSMEG_2064 Is Not Essential for the Viability of Mycobacteria, and Its Removal Causes Transcriptional Changes Related to the Induction of Nitrate Respiration

We applied homologous recombination to replace intact *rv3143* and its ortholog in *M. smegmatis msmeg_2064* with non-functional copies carrying large internal deletions. Then, the mutants were complemented with accessory copies of intact *rv3143*/*msmeg_2064* genes carrying the upstream sequences as putative promoters. The functionality of complementation was verified by qRT-PCR (*M. smegmatis*) or immunodetection (*M. tuberculosis*) (**Supplementary Figure S1**). The Western blotting analysis demonstrated no detectable level of Rv3143 protein in the Δ*rv3143* knockout *M. tuberculosis* strain and overproduction of Rv3143 in the complementing strain when compared to the control wild-type *M. tuberculosis* (**Supplementary Figure S1A**). qRT-PCR revealed the expected depletion of *msmeg_2064* gene expression in the mutant lacking the functional *msmeg_2064* gene and strong significant overexpression of *msmeg_2064* transcripts in the complementing strain compared to the control wild-type strain (**Supplementary Figure S1B**).

*M. tuberculosis* and *M. smegmatis* mutants defective in the synthesis of Rv3143/MSMEG_2064, as well as control wild-type strains grown in rich media, were examined for kinetics of growth by measuring the optical density of the culture at 600 nm (OD_600_) and viability by determining CFU/ml. The growth kinetics and viability of the mutants were not affected compared to those of the wild-type *M. smegmatis* (**Supplementary Figures S2A, B**) and *M. tuberculosis* strains (**Supplementary Figures S2C, D**). We concluded that Rv3143 and MSMEG_2064 are not essential for the growth and survival of mycobacteria propagated in a rich medium.

**Figure 3:**
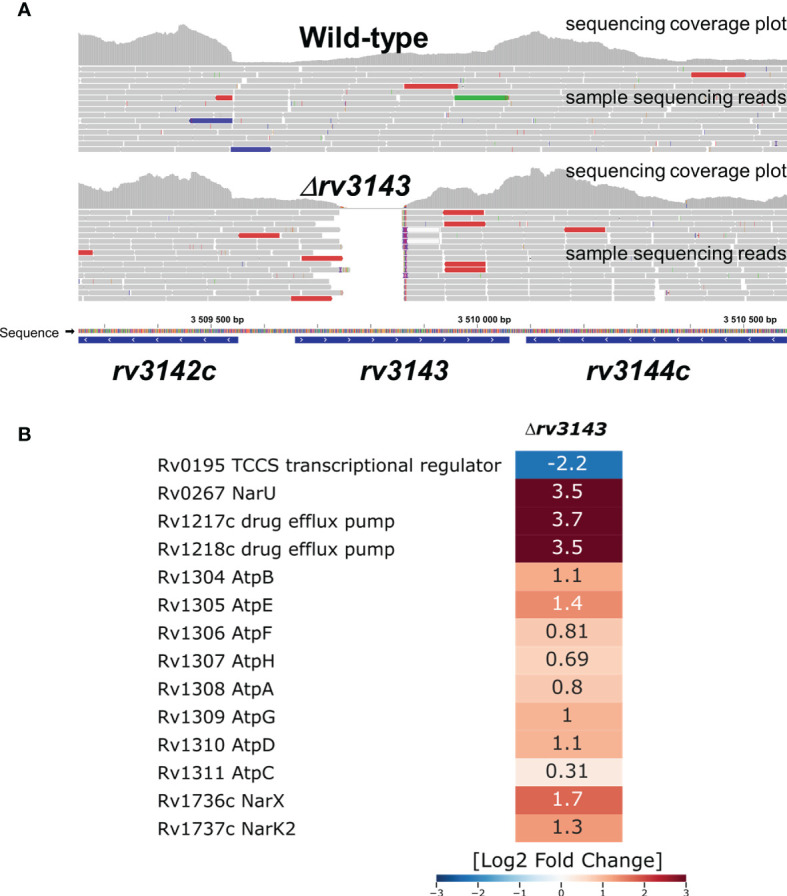
Selected transcriptional changes observed in the Δ*rv3143* mutant strain grown under standard laboratory conditions compared to the wild-type H37Rv *Mycobacterium tuberculosis* strain. **(A)** Confirmation of *rv3143* deletion from sequencing reads, image modified from Integrative Genomics Viewer v2.8.13. Regular paired-end sequencing reads are represented as gray bars. Colored reads either contain insertions (red) and deletions (blue) or have an opposite read pair orientation (green). **(B)** Log2-fold change values are shown in a heatmap generated in Seaborn software based on total RNA sequencing and differential expression estimation from the Degust online RNA-Seq analysis platform.

Total RNA sequencing revealed that depletion of Rv3143 in *M. tuberculosis* caused a reduction in expression of 34 genes and overexpression of 74 genes (Log2-fold change threshold set to ±1.58, with false discovery rate (FDR) <0.05) when compared to the wild-type strain grown under standard conditions (**Supplementary Table S2**). Interestingly, when the changes from RNA-Seq were inspected with the use of the transcription factor overexpression analysis tool TFOE spreadsheet from www.tbdb.com, the tool indicated a significant number of genes belonging to the putative NnaR regulon (Rv0260c). Among them, there were genes encoding proteins primarily involved in nitrogen metabolism and assimilation, particularly important since nitrogen is used by mycobacteria in the respiratory chain when oxygen availability is limited. The transcripts for NarU and NarX were overexpressed nearly 12 times and 3.5 times, respectively, and NarK2 was upregulated 2.5 times, falling just below the threshold level set in our analysis. A summary of the RNA sequencing analysis is shown in **Figure 3**. Deletion of Rv3143 also led to downregulation of the hypoxic TCSS regulator Rv0195 and overexpression of the Rv0601c and Rv0600c histidine kinases that cooperate with the TcrA transcriptional regulator. Other changes on the transcriptional level included overexpression of the mym operon (Rv3083-3089) and Rv3612c-3616c (RD1 region associated with ESX-1 system), whereas *prpCD* locus (Rv1128c-1129c, Rv1130-1131) was downregulated. Several changes in the levels of individual small regulatory RNAs were also noticeable, with ESX-associated ncrMT1234, ncRv3648c, ncRv1298, ASdes, and mcr16 found among the 10 most significantly upregulated transcripts.

### The MSMEG_2064-Defective Strain Is Sensitized to Reactive Nitrogen Species

**Figure 4:**
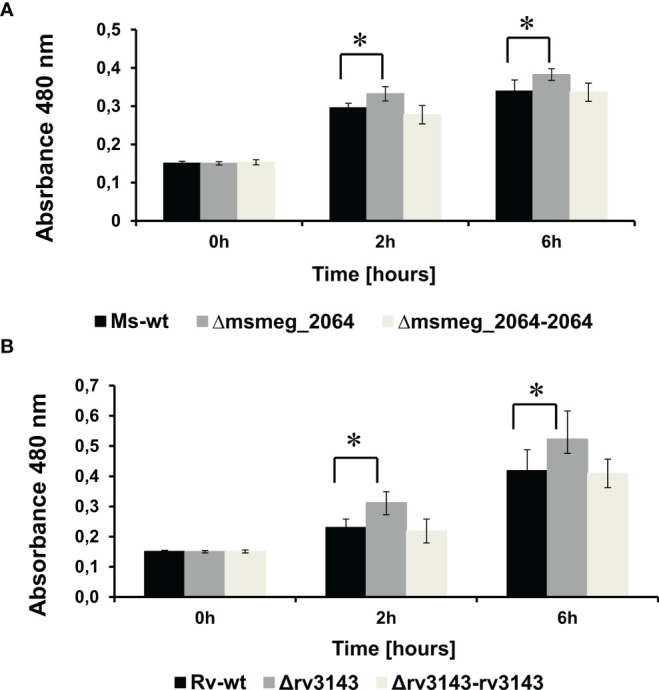
Measurement of reduction rates of a redox indicator—TTC—in Δ*msmeg_2064 Mycobacterium smegmatis* **(A)** and Δ*rv3143 Mycobacterium tuberculosis* **(B)** strains. Absorbance values at 480-nm values are the means ± SDs from three independent experiments. Statistical significance was determined using Student’s t-test **(A)** (^*^ *p* < 0.001); **(B)** (^*^ *p* < 0.001).

The interaction between Rv3143 and the Nuo complex might suggest that MSMEG_2064 and/or Rv3143 proteins affect the function of complex I of the respiratory chain in mycobacteria. To study the respiratory chain activity of the Δ*msmeg_2064* and Δ*rv3143* strains, we attempted to use TTC as an electron acceptor. The reduction of colorless TTC to red 1,3,5-triphenylformazan (TPF) was monitored by recording the absorbance at 480 nm. The potential defect in the entry point of electrons (Nuo) into the electron transport system could lead to a reduced cytosolic environment resulting in the reduced cytosolic TTC. Increased metabolic activity in the cells of the investigated mutants was observed. We noticed a modest but statistically significant increase in the utilization of TTC by approximately 12% in Δ*msmeg_2064* cells after 2 and 6 h of incubation at 37°C compared to the reduction in TTC measured in the wild-type cells (**Figure 4A**).

An increased level of TTC reduction (17% after 2 h and 20% after 6 h) was also observed in Δ*rv3143* cells compared to the control *M. tuberculosis* strain (**Figure 4B**). Furthermore, we observed the restoration of the phenotype to the wild-type strain of both complementing strains Δ*rv3143-attB::P_3143_rv3143* and Δ*msmeg_2064-attB::P_2064_msmeg_2064* encoding intact *rv3143* and *msmeg_2064* genes, respectively.

**Figure 5:**
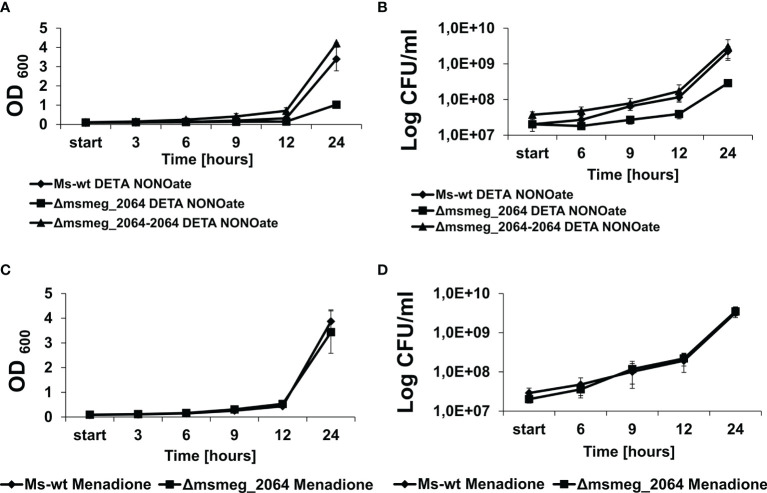
Kinetics of growth and viability of *Mycobacterium smegmatis* strains treated with DETA NONOate or menadione. The mutant Δ*msmeg_2064*, wild-type Ms-wt, and complementing Δ*msmeg_2064*–*2064* strains were grown in 7H9 Middlebrook-rich medium in the presence of reactive nitrogen (DETA NONOate) **(A, B)** and oxygen species (menadione) **(C, D)**. The growth of strains was evaluated by measuring the OD_600_ at the indicated time points **(A, C)**, and the numbers of viable cells were determined as bacterial colony-forming units (CFU) per ml on 7H10/OADC plates **(B, D)**. Means ± SDs are shown from three independent experiments.

Reactive nitrogen species treatment and hypoxia were reported to affect cellular respiration, causing a switch from the transcription of type I to type II NADH dehydrogenase and switching to nitrate respiration (Shi et al., 2005). Nitrate respiration is in fact believed to protect hypoxic mycobacteria against reactive nitrogen species (Tan et al., 2010). To evaluate the role of the MSMEG_2064 response regulator in the respiration process, we decided to examine the sensitivity of the investigated strains to reactive nitrogen (DETA NONOate 1,000 μM) and oxygen species (menadione 150 μM). In the presence of DETA NONOate, the growth of the Δ*msmeg_2064* mutant was significantly inhibited compared to that of the wild-type and complementation strains (**Figure 5A**). Additionally, the number of CFU was reduced by approximately 50% in the mutant exposed to DETA NONOate compared to the strains carrying an intact *msmeg_*2064 gene (**Figure 5B**). However, the inactivation of *msmeg_2064* did not affect the kinetics of *M. smegmatis* growth in the presence of reactive oxygen species generated by menadione or the viability CFU of the Δ*msmeg_2064* mutant compared to the control strains carrying an intact *msmeg_2064* gene (**Figures 5C, D**). We did not observe any significant difference in the survival of *M. tuberculosis* mutant in comparison to the wild-type strain upon exposure to DETA NONOate and menadione (**Supplementary Figure S3**).

### Deficiency of Rv3143 or MSMEG_2064 Affects the Membrane Redox Potential and Sensitivity to Valinomycin

To further investigate whether Rv3143/MSMEG_2064 affects the activity of complex I of the respiratory chain, we applied the microplate Alamar blue assay (MABA) to examine the sensitivity of the Δ*rv3143 M. tuberculosis* strain to compounds disrupting the electrochemical potential of membranes such as monensin and valinomycin. Monensin transports sodium ions through the membrane in an electrogenic and electroneutral manner. Valinomycin functions as a potassium-specific transporter and facilitates the movement of potassium ions through lipid membranes, disrupting the electrochemical potential gradient. We also tested the selected efflux pump inhibitors carbonyl cyanide *m*-chlorophenyl hydrazine (CCCP), an oxidative phosphorylation inhibitor, and trifluoperazine, which affect calcium-dependent ATPase. The strain defective in the synthesis of Rv3143 was not sensitized to monensin, CCCP, or trifluoperazine; however, the Δ*rv3143* mutant appeared to be 3 times more resistant to valinomycin (MIC_90_ 0.75 µg/ml) than the wild-type strain (MIC_90_ 0.25 µg/ml) (**Table 1**; **Supplementary Figure S4**).

**Table 1:** Minimum inhibitory concentration (MIC) of selected compounds used in these studies.

Selected compounds	MIC (μg/ml)	
	H37Rv	Δ*rv3143*
**Monensin**	3.125	3.125
**Valinomycin**	**0.25**	**0.75**
**CCCP**	5	5
**Trifluoperazine**	14	14
**Valinomycin+CCCP**	0.25	0.25
**Valinomycin+trifluoperazine**	0.25	0.25

The presented MIC values were obtained repeatable in three biological repeats. The bold numbers represent the difference in MIC value between control H37Rv and mutant strain.

However, the increased level of resistance to valinomycin observed in the Δ*rv3143* mutant was abolished in the presence of CCCP or trifluoperazine (**Table 1**). We additionally tested the sensitivity of the Δ*rv3143* mutant to the selected antituberculosis drugs, including ethionamide, isoniazid, capreomycin, ofloxacin, rifampicin, and streptomycin, which affect various molecular targets in the cells with no detectable differences in sensitivity compared to the wild-type strain (**Supplementary Table S3**).

### Deficiency of MSMEG_2064 in the Ndh Depletion Background Affects the Viability of *Mycobacterium smegmatis* Under Hypoxia and Reaeration Conditions

The transfer of electrons through the respiratory chain to the quinone pool in mycobacteria depends mainly on NADH dehydrogenases type II encoded by *ndh* (*M. smegmatis*) or *ndh* and *ndhA* (*M. tuberculosis*) (Yano et al., 2006). To visualize the potential modulatory effect of MSMEG_2064 on respiratory complex I, we decided to construct a Δ*msmeg_2064* mutant with downregulated expression of *ndh* by using the CRISPRi-dCas9 system. The recombinant *M. smegmatis* strains Δ*msmeg_2064-ndh^CRISPRi/dCas9^* and *ndh^CRISPRi/dCas9^* were analyzed with respect to the expression level of *ndh* by qRT-PCR compared to the control Δ*msmeg_2064* and wild-type carrying an “empty” CRISPRi/dCas9 vector (**Supplementary Figure S5**).

**Figure 6:**
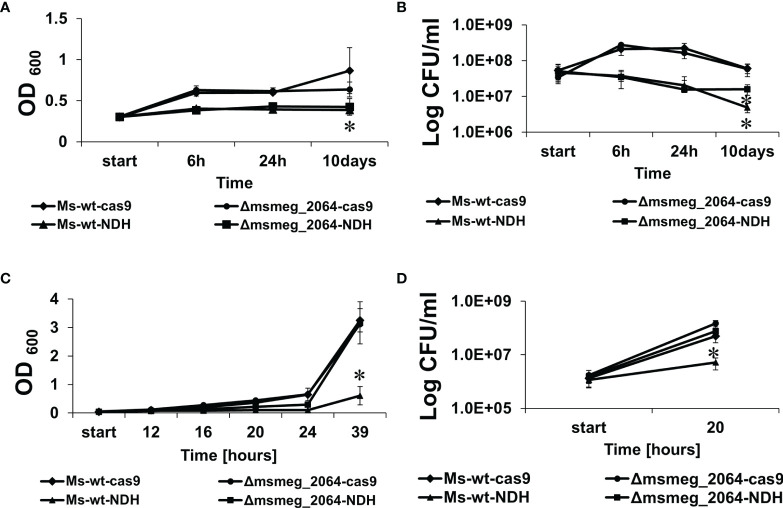
Kinetics of growth and viability of *Mycobacterium smegmatis* strains expressing decreased levels of *ndh* gene. The mutant strains *ndh* ^CRISPRi/dCas9^ (Ms-wt-NDH) and Δ*msmeg_2064-ndh* ^CRISPRi/dCas9^ (Δ*msmeg_2064*-NDH) and control strains CRISPRi/dCas9 (Ms-wt-cas9) and Δ*msmeg_2064* ^CRISPRi/dCas9^ (Δ*msmeg_2064*-cas9) were grown in 7H9/AD Middlebrook medium for 16 h at 37°C with the addition of anhydrotetracycline to deplete *ndh*. Then, bacteria were cultured in limited oxygen access for 10 days **(A, B)** and subjected to reaeration for 39 h (C,D). The growth of strains was evaluated by measuring the OD_600_ at the indicated time points (A,C), and the numbers of viable cells were determined as bacterial colony-forming units (CFU) per ml on 7H10/OADC plates (B, D). Mean ± SD from three independent experiments are shown. Statistical significance was determined using Student’s t-test **(A)** (^*^ *p* < 0.01); **(B)** (^*^ *p* < 0.0001); **(C)** (^*^ *p* < 0.001); **(D)** (^*^ *p* < 0.0001).

Both mutants carrying *ndh^CRISPRi/dCas9^* appeared to express between 5 and 10 times less mRNA carrying information for *ndh* as compared to the control strains. The constructed mutants and control strains were grown under hypoxia and reaeration conditions as described in the *Materials and Methods*. In the absence of aTc (inducing CRISPRi/dCas9 expression), with an unaffected expression of *ndh*, the kinetics of the growth and survival of mutants were not different as compared to the control strains (**Supplementary Figures S6A, B**). However, growth inhibition was observed for Δ*msmeg_2064-ndh^CRISPRi/dCas9^* and *ndh^CRISPRi/dCas9^* mutants if aTc was supplemented into the media (**Figure 6A**).

CFU analysis revealed a significant decrease in the number of viable cells of both mutants depleted of Ndh compared to the controls (**Figure 6B**). Moreover, after 10 days of growth under hypoxic conditions, the number of viable cells of the *ndh^CRISPRi/dCas9^* mutant decreased significantly more (−11.3-fold change vs. wild type carrying the CRISPRi/dCas9 vector, *p* < 0.001) than the number of viable cells of the Δ*msmeg_2064-ndh^CRISPRi/dCas9^* mutant defective in the synthesis of MSMEG_2064 and depleted of Ndh (−2.64-fold difference vs. Δ*msmeg_2064* carrying the CRISPRi/dCas9 vector, *p* < 0.001; +2.24-fold difference vs. *ndh^CRISPRi/dCas9^* mutant, *p* < 0.001).

Furthermore, to reactivate the bacilli, the oxygen-depleted cultures were diluted in 7H9/AD rich medium and incubated at 37°C with intensive shaking for 39 h in conditions with free access to oxygen. All cultures not supplemented with aTc presented no differences in growth and viability after reaeration (**Supplementary Figures S6C, D**). However, the depletion of Ndh significantly inhibited the growth of the *ndh^CRISPRi/dCas9^* mutant but not the mutant defective in the synthesis of MSMEG_2064 with depleted Ndh (Δ*msmeg_2064-ndh^CRISPRi/dCas9^*). Moreover, the number of viable bacilli detected 20 h after reaeration decreased significantly (9-fold, *p* = 0.005) in the *ndh^CRISPRi/dCas9^* mutant compared to the control strains and in the Δ*msmeg_2064-ndh^CRISPRi/dCas9^* mutant (**Figures 6 C, D**). We did not observe the effect of hypoxia on the efficacy of CRISPR system. The qRT-PCR confirmed the downregulation of *ndh* expression under hypoxic conditions (24 h and 10 days) in *M. smegmatis ndh^CRISPRi/dCas9^* and Δ*msmeg_2064-ndh^CRISPRi/dCas9^* strains compared to the control strain carrying an “empty” CRISPRi/dCas9 vector (**Supplementary Figures S7A, B**).

### Depletion of Ndh But Not Inactivation of *msmeg_2064* Affects the Expression Level of *nuoA* and *nuoD*

**Figure 7:**
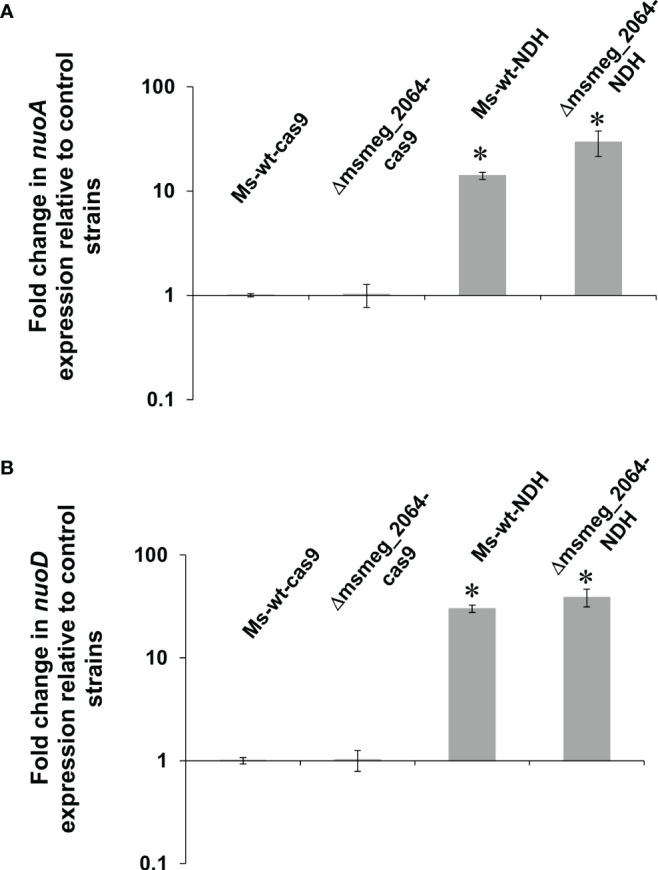
Expression profile of *nuoA* **(A)** and *nuoD* **(B)** genes in *Mycobacterium smegmatis* strains expressing depleted *ndh*. Mutant strains *ndh* ^CRISPRi/dCas9^ (Ms-wt-NDH) and Δ*msmeg_2064-ndh* ^CRISPRi/dCas9^ (Δ*msmeg_2064*-NDH) and control strains CRISPRi/dCas9 (Ms-wt-cas9) and Δ*msmeg_2064* ^CRISPRi/dCas9^ (Δ*msmeg_2064*-cas9) were grown in 7H9/AD broth for 16 h at 37°C with the addition of anhydrotetracycline. Transcript levels were determined using qRT-PCR and SYBR green chemistry. The expression levels of *nuoA*/*nuoD* were normalized to the *sigA* housekeeping gene and compared to the control strain. Statistical significance was determined using Student’s t-test **(A)** (^*^ *p* < 0.001); **(B)** (^*^ *p* < 0.001).

NDH-2 in *M. tuberculosis* is conditionally essential and might be compensated by the activity of the NDH-1 complex. We asked the question about the expression level of the selected proteins of the Nuo complex in the Ndh depletion genetic background. The expression levels of *nuoA* and *nuoD* genes were analyzed in the *M. smegmatis* mutant with inducible depletion of Ndh (*ndh^CRISPRi/dCas9^*), in the mutant defective in the synthesis of MSMEG_2064 (Δ*msmeg_2064*), and the double-mutant Δ*msmeg_2064-ndh^CRISPRi/dCas9^*. Both analyzed genes were significantly overproduced (14-fold increase in *nuoA* and 30-fold increase in *nuoD* transcript levels) in both mutants with depleted Ndh compared to the control strain (wild-type strain carrying an “empty” CRISPRi/dCas9 vector). The expression of the investigated *nuo* genes was not affected at the transcriptional level by the inactivation of *msmeg_2064* (**Figure 7**).

### MSMEG_2064 Deficiency in the Ndh Depletion Background Affects Oxygen Consumption

The oxygen consumption in the culture was monitored by discoloration of methylene blue as an indicator. We compared the dynamics of oxygen consumption in the culture of *M. smegmatis* mutants defective in the synthesis of MSMEG_2064 (Δ*msmeg_2064*) and strains with inducible depletion of Ndh (*ndh^CRISPRi/dCas9^*, Δ*msmeg_2064-ndh^CRISPRi/dCas9^*) in comparison to the control wild-type strain with or without an “empty” *CRISPRi/dCas9* vector as well as Δ*msmeg_2064* complemented with an intact *msmeg_2064* gene (*P_tet_-msmeg_2064*). All strains growing without aTc as an inducer of Ndh depletion presented a similar time of methylene blue discoloration as was monitored by OD_600_ readings. In the presence of aTc, oxygen consumption was dramatically slowed down in the *ndh^CRISPRi/dCas9^* mutant (6 vs. 12 h compared to the control). The inactivation of *msmeg_2064* in the Ndh depletion background further delayed oxygen consumption by approximately 2 h (**Table 2**).

**Table 2:** Monitoring of oxygen consumption by methylene blue discoloration under hypoxic conditions.

Strain	i	3 h	4 h	5 h	8 h	9 h	10 h	11 h	12 h	13 h
Ms-wt	−	0.609 ± 0.023	0.363 ± 0.084	0.067 ± 0.008						
Δ*msmeg_2064*	−	0.626 ± 0.023	0.578 ± 0.052	0.085 ± 0.002						
Δ*msmeg_2064*::2064	−	0.636 ± 0.045	0.558 ± 0.034	0.093 ± 0.006						
Ms-wt CRISPRi/dCas9	−	0.632 ± 0.031	0.432 ± 0.165	0.055 ± 0.017						
Δ*msmeg_2064*CRISPRi/dCas9	−	0.419 ± 0.191	0.18 ± 0.025	0.045 ± 0.028						
Ms-wt-ndhCRISPRi/dCas9	−	0.636 ± 0.008	0.287 ± 0.063	0.048 ± 0.000						
Δ*msmeg_2064*ndhCRISPRi/dCas9	−	0.184 ± 0.042	0.108 ± 0.077	0.031 ± 0.002						
Ms-wtCRISPRi/dCas9	+	0.647 ± 0.047	0.312 ± 0.279	0.065 ± 0.028						
Δ*msmeg_2064*CRISPRi/dCas9	+	0.213 ± 0.034	0.144 ± 0.076	0.017 ± 0.005						
Ms-wt-ndhCRISPRi/dCas9	+				0.629 ± 0.002	0.624 ± 0.004	0.435 ± 0.167	0.085 ± 0.005		
Δ*msmeg_2064*ndhCRISPRi/dCas9	+				0.554 ± 0.032	0.622 ± 0.012	0.476 ± 0.196	0.516 ± 0.066	0.278 ± 0.052	0.065 ± 0.005

The results are the absorbance readings at 600 nm and mean ± SD from three independent experiments. The addition of anhydrotetracycline as an inducer of CRISPRi/Cas9-driven gene expression silencing is indicated in the “i” column.

## Discussion

The bioinformatics analyses of the Rv3143 protein, annotated as an “orphan” regulatory protein of the two-component regulatory system, indicate the presence of an N-terminal receiving domain REC and the absence of nucleic acid-binding domains in its structure. As reported, 70% of regulatory proteins contain the DNA binding domain and act as transcription regulators (Zschiedrich et al., 2016). Moreover, the Rv3143 protein was not phosphorylated by selected histidine kinases belonging to the already known TCSSs, and under the influence of acetyl phosphate, the compound presents a high potential for transferring phosphate groups (Agrawal et al., 2015). Hence, we postulated that Rv3143 acts as an auxiliary protein or an intermediary binding to other proteins. Importantly, Rv3143 was previously found to be overexpressed in multidrug-resistant tuberculosis strains, along with the DevR, MtrA, and RegX3 two-component system regulators (Zhou et al., 2015).

The aim of this study was to determine the role of the Rv3143 response regulator in the physiology of *M. tuberculosis*. Due to the high pathogenicity and slow growth of *M. tuberculosis*, part of this work was performed in its fast-growing, saprophytic cousin, *M. smegmatis*. To investigate the function of Rv3143, we decided to search for proteins interacting with the putative response regulator. Expression of Rv3143 protein fused with GFP as bait and analysis of purified protein complexes by MS/MS allowed identification of potential partner proteins for Rv3143 as soluble protein components of NADH dehydrogenase I, Nuo, or complex I: NuoB, NuoC, NuoD, NuoE, NuoF, NuoG, and NuoI. The MS analyses, repeated in the presence of a crosslinking agent, confirmed direct interactions of Rv3143 with proteins belonging to the Nuo complex and indicated NuoD as the most likely direct partner of Rv3143. The interaction between Rv3143 and NuoD was confirmed further by pull-down assays and was screened for direct interactors using MS facilitated by BS3 crosslinking. The pair of NuoD and Rv3143 had the highest number of crosslinks identified by Kojak software, which suggests the location of Rv3143 in respiratory complex I and its role in oxidative phosphorylation. It is interesting that in actinobacteria, the orthologs of *rv3143* gene are located in direct or very close proximity to the operon of *nuo* genes, encoding NuoA–N subunits of NADH dehydrogenase type I. The CheY protein, which is the closest equivalent of the Rv3143 protein in *E. coli*, possesses a phosphate-binding domain in its structure, but similarly to Rv3143, it does not have the effector domain. The CheY protein is an element of the system responsible for controlling flagella in the chemotaxis process, and when phosphorylated, it binds to the FliM protein, which then interacts with the NuoCD protein. Additionally, flagella, requiring energy for their movement, associate with the oxidative phosphorylation chain in the cell membrane (Rajagopala et al., 2007; Zarbiv et al., 2012). These findings may suggest that while the flagellar system was reduced in mycobacteria resulting in the loss of FliM protein, the link between CheY-like regulator and the Nuo complex, and NuoD protein, in particular, was sustained for other purposes, possibly oxygen sensing, redox sensing, or related sensing mechanisms. The majority of crosslinks found between the Rv3143 protein and NuoD appeared in the amino acid insertion region of NuoD, which does not occur in *Proteobacteria*. Importantly, the presence of this additional stretch of amino acids is specific to actinobacteria and allows their separate clustering in phylogenetic analysis. We used a collection of bacterial NuoD proteins to perform such analysis, and upon marking the co-occurrence of Rv3143 orthologs in the investigated species, it became very clear that there is a correlation between NuoD clustering and the presence of the response regulator we investigated.

As expected based on high-density transposon mutagenesis (Sassetti et al., 2003; DeJesus et al., 2017), Rv3143 and its ortholog in *M. smegmatis* (MSMEG_2064) were not essential for viability, and the genes encoding these proteins could be replaced with non-functional forms. The constructed mutants and control strains were analyzed under various conditions of growth. In the presence of reactive nitrogen species generated by DETA NONOate, the Δ*msmeg_2064* mutant exhibited a significant reduction in growth kinetics and viability compared to the wild-type strain. Free radicals generated by DETA NONOate under standard laboratory conditions were previously reported to have minor bacteriostatic effects on clinical *M. tuberculosis* strains (Voskuil et al., 2011). Interestingly, tubercle bacilli under the influence of reactive nitrogen species in anaerobic conditions inhibit the transcription of NADH dehydrogenase type I *in vitro* while inducing the transcription of NADH type II. Under similar conditions but *in vivo*, fourfold repression of *nuoB* gene, encoding the subunit of NDH-1 dehydrogenase, was observed (Shi et al., 2005). Similarly, we noticed a correlation between the depletion of Rv3143 and the expression of NarU, NarK2, and NarX, which was overexpressed in the mutant strain lacking Rv3143 altogether. All three nitrogen metabolism enzymes listed above are postulated to be crucial to nitrate respiration in *M. tuberculosis* (Sohaskey and Wayne, 2003; Huang et al., 2015) and are typically overexpressed during hypoxia (Kundu and Basu, 2021). In contrast, Nuo complex proteins are typically downregulated under hypoxic conditions, when nitrate respiration is switched on, and are overexpressed during growth reactivation, when bacterial cultures are switched back to oxygen-rich growth conditions (Kundu and Basu, 2021). This may suggest that even though the Nuo complex is not the dominant NADH dehydrogenase in mycobacteria, bacilli lacking Rv3143 activate alternative respiratory mechanisms, and the Nuo complex with Rv3143 may be critical to oxygen sensing and downstream signaling.

To further characterize the biological effect of the identified interactions between Rv3143 and respiratory complex I, we further investigated the sensitivity of the Δ*rv3143* strain to the selected compounds disrupting the oxidative phosphorylation process and the membrane potential. While testing the sensitivity of bacterial strains to monensin, valinomycin, CCCP, and trifluoperazine, we noticed a threefold increase in the resistance of the Δ*rv3143* mutant to valinomycin. However, the observed increased resistance to valinomycin was abolished to the wild-type level in the presence of CCCP and trifluoperazine. Valinomycin reduces the natural electrochemical potential of the cell, decoupling the processes of oxidative phosphorylation. Valinomycin is highly selective for potassium cations, which are transported through cell membranes. CCCP inhibits the activity of the proton pump by reducing the membrane potential and inhibits oxidative phosphorylation, which causes a decrease in the activity of ATP synthase (Pule et al., 2016). The addition of the protonophore CCCP to *M. tuberculosis* strains resistant to ofloxacin has previously been reported to increase their sensitivity, suggesting the importance of proton pumps for resistance to fluoroquinolones (Singh et al., 2011; Sun et al., 2014). Moreover, Gupta and colleagues observed decreased resistance to rifampicin, isoniazid, and streptomycin in *M. tuberculosis* cells cultured in the presence of CCCP (Gupta et al., 2006). CCCP at higher concentrations affects not only the transport of hydrogen cations but also other ions, including potassium ions (Rao et al., 2001). In turn, trifluoperazine belongs to the class of phenothiazine compounds and shows a pleiotropic effect in mycobacteria, affecting the synthesis of lipids and proteins, DNA processes, and respiration, including calcium-dependent ATPases or inhibition of type II NADH dehydrogenase (Advani et al., 2012; Black et al., 2014). As reported in *M. tuberculosis*, trifluoperazine inhibits growth and reduces resistance to rifampicin, contributing to the elimination of mycobacteria residing in macrophages (Crowle et al., 1992; Reddy et al., 1996). Our findings suggest that the Rv3143 interaction with the Nuo complex may interfere with resistance to antibiotics and reactive radicals in mycobacteria.

We then decided to determine the metabolic activity of the Δ*msmeg_2064*/Δ*rv3142* mutants using the known electron acceptor TTC. The assessment of TTC reduction by microorganisms is exploited for screening for cells that have a dysfunctional respiratory chain (Rich et al., 2001). The observed increased level of reduction of TTC by the mutant cells confirmed a potential defect in the electron transport system. In contrast, Dadura and colleagues (2017) reported a decreased level of reduction in TTC, indicating a slowdown in the respiration process in the Δ*pdtaS M. smegmatis* mutant lacking the functional histidine kinase PdtaS. Mutants of Δ*pdtaS* exhibited altered susceptibility to aminoglycoside antibiotics targeting 30S ribosomes as well as to tetracycline (Dadura et al., 2017).

As previously reported elsewhere, the *nuo* operon is not essential for the growth and survival of mycobacteria *in vitro* under hypoxic conditions (Rao et al., 2008; Vilchèze et al., 2018). Limited oxygen access leads to the overproduction of dehydrogenase type II, Ndh, and cytochrome oxidase bd and a reduction in the production of NADH dehydrogenase type I (Schubert et al., 2015; Prosser et al., 2017). In addition to respiratory complex I (NDH-1), *M. tuberculosis* transfers electrons to quinone by NADH dehydrogenases type II (NDH-2), which are encoded by *ndh* and *ndhA* genes (Yano et al., 2006). Fast-growing *M. smegmatis* possesses only a single copy of NDH-2 (Ndh), but it represents 95% of the total NADH oxidation measured (Vilchèze et al., 2005). Therefore, we decided to extend our research by demonstrating the role of the Rv3143 protein in the respiration process and examining the phenotypic effect of the lack of MSMEG_2064 protein in a strain with a silenced expression of *ndh* gene coding for NADH type II dehydrogenase. Downregulation of *ndh* expression was achieved using the CRISPR-Cas system. Studies on the growth kinetics and survival of mutant strains under conditions of limited access to oxygen revealed a slowdown in the rate of growth and a significant decrease in the viability of strains with silenced *ndh* genes compared to control strains. After hypoxia, the tested strains were reactivated by changing the growth conditions from anaerobic to aerobic. We observed a significant slowdown in the growth kinetics and decreased viability in mutants expressing the functional *msmeg_2064* gene and silenced *ndh.* In the reaeration process, the inhibition of growth and decreased viability were observed for mutants with depleted Ndh; however, the inactivation of *msmeg_2064* reversed this effect to the level of the control strain. Homology search databases such as KEGG recognize the Rv3143 protein as an ortholog of the response regulator domain of eubacterial aerobic respiration sensor-response proteins (ArcAs), and we think that oxygen sensing is the most plausible function of Rv3143 in mycobacteria. The ArcAB oxygen sensing system is not present in mycobacteria, and its function is fulfilled by other regulators, such as SenX-RegX3 (Singh and Kumar, 2015), and possibly other proteins. While the mechanisms of oxygen sensing are enormously important to the physiology and survival of this intracellular pathogen, they are not yet completely understood.

Our study sheds light on the intracellular function of the Rv3143 protein, which influences the efficiency of the respiratory chain in *Mycobacterium* and controls the nitrate respiration switch in this bacterium. However, the precise mechanism of action of the protein studied here requires further detailed analysis to fully understand the above-described phenomena.

## Data Availability Statement

The datasets presented in this study can be found in online repositories. The names of the repository/repositories and accession number(s) can be found in the article/**Supplementary Material**. The RNA-seq related data have been deposited to the GEO database and are accessible at https://www.ncbi.nlm.nih.gov/geo/query/acc.cgi?acc=GSE193950.

## Ethics Statement

The animal study was reviewed and approved by Polish Local Ethics Commission for Experiments on Animals No. 9 in Lodz (Agreement 54/ŁD1/2011).

## Author Contributions

RP: conceptualization, investigation, visualization, writing—original draft, and funding acquisition. KW: investigation. PP: conceptualization, investigation, visualization, and writing. EL: investigation. MA: investigation. EB: investigation. BD: investigation. MS: investigation. AR-G: investigation. JD: conceptualization, writing—review and editing, and supervision. All authors listed have made a substantial, direct, and intellectual contribution to the work and approved it for publication.

## Funding

This work was supported by the National Science Centre, Republic of Poland, SONATA 6—2013/11/D/NZ6/02888 (to RP) and by Foundation for Polish Science, PARENT-BRIDGE Program, co-financed by the European Union with European Regional Development, BRIDGE/2013-8/10 (to RP).

## Conflict of Interest

The authors declare that the research was conducted in the absence of any commercial or financial relationships that could be construed as a potential conflict of interest.

## Publisher’s Note

All claims expressed in this article are solely those of the authors and do not necessarily represent those of their affiliated organizations, or those of the publisher, the editors and the reviewers. Any product that may be evaluated in this article, or claim that may be made by its manufacturer, is not guaranteed or endorsed by the publisher.
