# Supplementary material for: The Orphan Response Regulator Rv3143 Modulates the Activity of the NADH Dehydrogenase Complex (Nuo) in Mycobacterium tuberculosis via Protein–Protein Interactions
Source: Front Cell Infect Microbiol. 2022 Jun 28;12:909507. doi: 10.3389/fcimb.2022.909507 (PMC9274095; doi:10.3389/fcimb.2022.909507)
Supplement: Supplementary file 1 [file DataSheet_1.pdf]

Supplementary materials

The orphan response regulator Rv3143 modulates the activity of the NADH dehydrogenase complex (Nuo) in *Mycobacterium tuberculosis* via protein-protein interactions

Renata Płocińska[1], Karolina Wasik[1], Przemysław Płociński[1,2], Ewelina Lechowicz[1], Magdalena Antczak[1], Ewelina Błaszczyk[1], Bożena Dziadek[3], Marcin Słomka[4], Anna Rumijowska-Galewicz[1], Jarosław Dziadek[1*]

[1] Institute of Medical Biology of the Polish Academy of Sciences, Lodowa 106, 93-232 Łódź, Poland

[2] Department of Immunology and Infectious Biology, Faculty of Biology and Environmental Protection, University of Łódz, Banacha 12/16, 90-237 Łódź, Poland

[3] Department of Molecular Microbiology, Faculty of Biology and Environmental Protection, University of Łódz, Banacha 12/16, 90-237 Łódź, Poland

[4] Biobank Lab, Department of Molecular Biophysics, Faculty of Biology and Environmental Protection, University of Łódź, Pomorska 139, 90-235 Łódź, Poland

* Correspondence should be addressed to jdziadek@cbm.pan.pl

Running title: Rv3143 fine-tunes the activity of the NADH dehydrogenase complex in mycobacteria via protein-protein interactions.

Keywords: tuberculosis, oxidative respiration, orphan two-component regulators, signal transduction, respiratory chain, NADH dehydrogenase

Table of contents:

1. **Table S1a:** List of proteins identified as possible interaction partners for Rv3143 (spreadsheet in a separate excel file associated with this manuscript)
2. **Table S1b:** List of protein-protein crosslinks identified between the Rv3143 and Nuo proteins (spreadsheet in a separate excel file associated with this manuscript)
3. **Table S2:** Genes differentially expressed between ΔRv3143 and wild type strains grown exponentially in 7H9 media supplemented with OADC (separate excel file associated with this manuscript)
4. **Table S3:** Minimum inhibitory concentration (MIC) of antibiotics used in these studies.
5. **Table S4:** List of strains, plasmids and primers used in this study.
6. **Supplementary Figures with corresponding legends.**
7. **Original blots for figure 2.**
8. **References**

Table S3. Minimum inhibitory concentration (MIC) of antibiotics used in these studies.

**Selected antibiotics**	**Minimum inhibitory concentration (MIC) (μg/ml)**	
	**H37Rv**	**Δ*rv3143***
**Ethionamide**	100	100
**Isoniazid**	0,0625	0,0625
**Capreomycin**	2,5	2,5
**Ofloxacin**	0,5	0,5
**Rifampicin**	0,001	0,001
**Streptomycin**	1,0	1,0

Table S4

A. Primers used in this study		
Name of primer	Sequence (5'>3')	Application
	**Primers used to amplify DNA for targeted gene replacement**	
Msmeg_2064GR1ScaI-F	CAGTACTGATGTCGCGAACCGCTTGTT	*msmeg_2064* knock-out
Msmeg_2064GR2HindIII-R	CAAGCTTTACACAAGGATGCGCAGCGC	
Msmeg_2064GR3HindIII-F	CAAGCTTTGGCGATCCTCGACGGCG	
Msmeg_2064GR4NotI-R	GCGGCCGCTTCGTCACCGCGGGGCAG	
Msmeg_2064BglII-F	CAGATCTATGGCCGGCTCCGCGCCGCT	Southern blotting - hybridization probe
Msmeg_2064XbaI-R	TCTAGAGCTACTGAGCGGGTGCCCGCAGCA	
Rv3143GR1PstI-F	ACTTCGCGCTGGCGGTCCG	*rv3143* knock-out
Rv3143GR2HindIII-R	CAAGCTTGCCCAGGGCCCGCATCAC	
Rv3143GR3HindIII-F	CAAGCTTCGGAGGCATGGGAATCGC	
Rv3143GR4NotI-R	GCGGCCGCGGAAGGACGGGAGCTGTG	
Rv3143EcoRI-F	CGAATTCGTGCCCGACTCCAGCACCGC	Southern blotting - hybridization probe
Rv3143HindIII-R	CAAGCTTAGTGGGCGGGTGCGCGC	
	**Primers used for clonings**	
Msmeg_2064-P-XbaI-F	CTCTAGATCGTCATCAGCGCGGCGT	*msmeg_2064* complementation
Msmeg_2064-P-HindIII-R	CAAGCTTAACGGCGGCAGCGTGATTC	
Rv3143-P-XbaI -F	CTGTAGAGCGAAATCGAAGAGCGGACG	*rv3143* complementation
Rv3143-P-HindIII-R	CAAGCTTCAAATGCGGGGCGGCTGAA	
Rv3143GFP-F	GCTCGCTACTCTCATCGTGGAATCCTGACAGGACCCCAGGGAGGAAGCCGAACGATGCCCGACTCCAGCACC	Rv3143 fused with GFP (MS/MS)
Rv3143GFP-R	CTTGTACAGCGAGGTGATGTCGGCGGCCTTAAGCTTGTGGGCGGGTGCGCGCAA	
Rv3143GSTBglII-F	CAGATCTGTGCCCGACTCCAGCACCGC	Rv3143 overproduction
Rv3143GSTEcoRI-R	CGAATTCTTAGTGGGCGGGTGCGCGC	
NuoD-F	GGCTGTCTTAGAACAGTTAGGAGGTATGACGCAATCGCCGACTCG	NuoD overproduction
NuoD-R	CGGAGCTCGAATTXGGATCCTACCGGTCCACCCCGCCCATGA	
	**Primers used for qRT_PCR**	
Msmeg_2064RT-F	CGCTGCGCATCCTTGTGTACAG	*msmeg_2064* transcripts
Msmeg_2064RT-R	TCGTCCTTGACCTGCTTGGCC	
Msmeg_ndh-F	AAGTAGGACTGACCCGCGCCC	*msmeg_3621* transcripts
Msmeg_ndh-R	CACCTCTTCCAGCCGCTGCTC	
Msmeg_nuoA-F	CGATCGCGGCCGTTTTCG	*msmeg_2063* transcripts
Msmeg_nuoA-R	CGCCCACGGGTACAGAAAGACG	
Msmeg_nuoD-F	AACATGGGCCCGCAGCATC	*msmeg_2060* transcripts
Msmeg_nuoD-R	TGAGCATCACGCGGATCACG	
	**Primers used for strains with controlled depletion**	
Msmeg_ndh_sPAM_S	GGGAAGATCGATGTGCGTCACATCGC	Oligonucleotide cloned into pLJR962
Msmeg_ndh_sPAM_R	AAACGCGATGTGACGCACATCGATCT	

		vector (ndh silencing)
**B. Strains constructed for this study**		
Name	Description	Reference
	**STRAINS**	
Top10F’	*Escherichia coli* strain	Invitrogen
Mc^{2}155	*M. smegmatis* wild type	Laboratory stock
BL21 (DE3)	*E. coli* strain	Invitrogen
H37Rv	*M. tuberculosis* wild type	Laboratory stock
Δ*msmeg_2064*	*M. smegmatis msmeg_2064* deletion strain	This study
Δ*msmeg_2064*::$_{pmsmeg\_2064}$*msmeg_2064*	*M. smegmatis msmeg_2064* deletion strain carrying complementation plasmid	This study
Δ*rv3143*	*M. tuberculosis rv3143* deletion strain	This study
Δ*rv3143*:: $_{prv3143}$*rv3143*	*M. tuberculosis rv3143* deletion strain carrying complementation plasmid	This study
ndh$^{CRISPRi/dCas9}$	*M. smegmatis* wild-type strain with down-regulated expression of *ndh*	This study
Δ*msmeg_2064-ndh*$^{CRISPRi/dCas9}$	*M. smegmatis* strain defective in the synthesis of MSMEG_2064 with depleted Ndh	This study
**Plasmids used for this study**	**CLONING VECTORS**	
pJET 1.2/blunt	Blunt cloning vector, Amp^R	Thermo Scientific
p2NIL	Recombination vector, nonreplicating in mycobacteria, Kan^R	Parish & Stoker, 2000 (1)
pGoal17	Source of PacI cassette, Amp^R	Parish & Stoker, 2000 (1)
pMV261	Mycobacterial replicating vector carrying heat shock hsp60 promoter, Kan^R	Med-Immune Inc
pMV306K	Mycobacterial integrating vector, Kan^R	Med-Immune Inc
pKW08-eGFP	Integrating vector carrying gene coding eGFP and tetracycline inducible promoter, Hyg^R	Plocinski, P. et al., 2014 (2)
pKW08Lx	Mycobacterial replication vector carrying inducible P_{tet} promoter, Hyg^R	Laboratory stock Robertson el., 2010 (3)
pGEX-6P-2	Bacterial expression vector enabling the purification of GST-tagged proteins, Amp^R	Amersham Biosciences
pE-SUMO	Expression vector with T7 promoter, allows the expression of recombinant proteins in fusion with a small ubiquitin-like protein and a histidine tag, Kan^R	LifeSensors Inc.
pLJR962	Mycobacterial vector carrying tetracycline inducible repressor, enabling the regulated gene silencing, Kan^R	Rock, J. et al., 2017 (4)
pKW3	1020 bp upstream fragment of *msmeg_2064* gene cloned in p2Nil vector, Kan^R	This study
pKW4	Δ *msmeg_2064* and its flanking regions cloned in p2Nil vector, Kan^R	This study
pKW5	pKW4 carrying PacI cassette, Kan^R	This study
pRD155	*msmeg_2064 gene* cloned into pKW08Lx replicative vector, carrying P_{tet} promoter, Hyg^R	This study
pKW8	1268 bp upstream fragment of *rv3143* gene cloned in p2Nil vector, Kan^R	This study

pKW9	Δ *rv3143* and its flanking regions cloned in p2Nil vector, Kan^R	This study
pKW10	pKW9 carrying PacI cassette, Kan^R	This study
pKW14	*rv3143* gene cloned into pGEX-6P-2 vector, Amp^R	This study
pKW15	*nuoD* gene cloned into pE-SUMO vector, Kan^R	This study
pKW16	pLJR962carrying 20 bp fragment of *ndh* gene, cloned into BsmBI restriction site, Kan^R	This study
pKW17	*rv3143* gene cloned into pKW08-eGFP vector, Hyg^R	This study
pKW18	*rv3143* gene cloned into pMV306 integrating vector, Kan^R	This study

Figure S1.

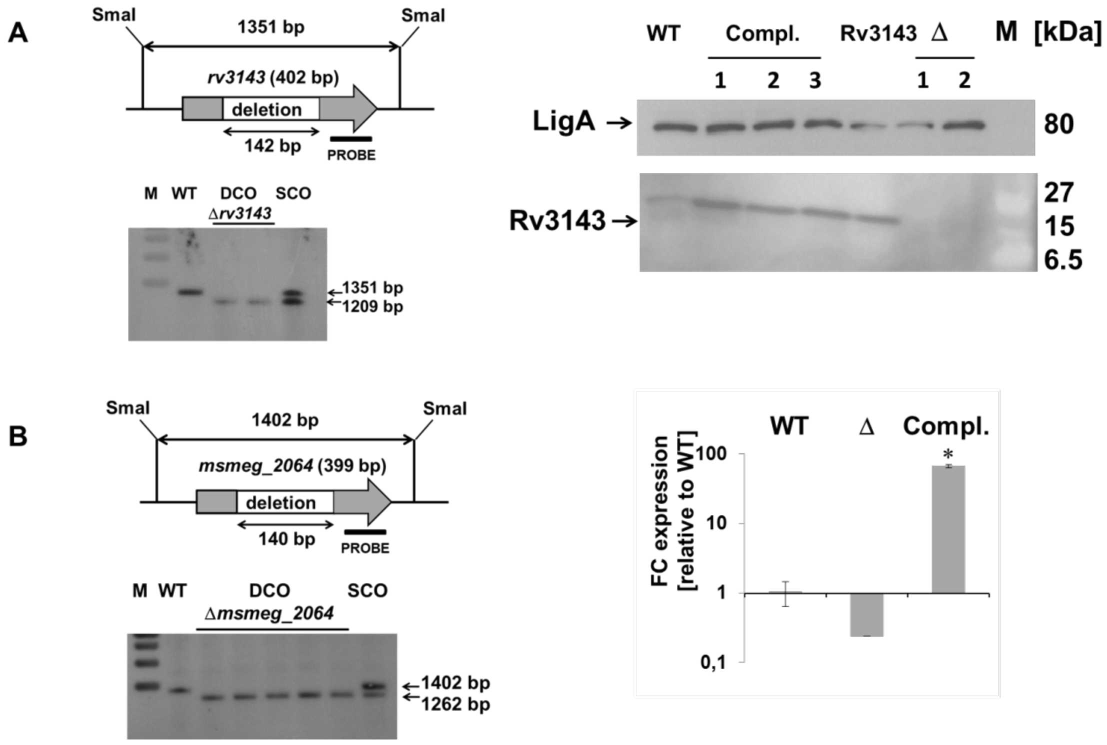

Figure S1. Construction and confirmation of deletion mutant strains: *M. tuberculosis* lacking rv3143 (A) or its ortholog msmeg_2064 in *M. smegmatis* (B). Southern blotting results are shown on the left-hand side panel for each strain, with an associated map of the genomic fragment in the vicinity of the deleted gene. Western blot immunodetection representing Rv3143 levels in *M. tuberculosis* cells is shown (A, right-

hand side panel), for which 20 µg of protein from the cellular lysates was resolved on SDS-PAGE gel, transferred to PVDF membranes and probed with mouse polyclonal anti-Rv3143 or rabbit polyclonal anti-LigA antibodies. WT – wild-type strain, Δ - mutant strain, Comp – complementing strain, Rv3143 – strain producing elevated Rv3143 levels (P_{tet}::*rv3143*). For the *M. smegmatis* mutant Δ*msmeg_2064*, complementing strain Δ*msmeg_2064-2064* and the wild type, gene deletion and complementation were confirmed by real-time PCR. Total RNA from three independent bacterial cultures was isolated, and the transcript levels were determined by applying qRT-PCR and SYBR green chemistry. The expression levels of *msmeg_2064* were normalized to the *mysA* housekeeping gene and compared to the control strain. Statistical significance was determined using Student's t-test ($*$ p $<$0,001).

Uncropped versions of figure panels

Figure S1

Southern blot confirmation of deletion of *M. tuberculosis* mutant strains

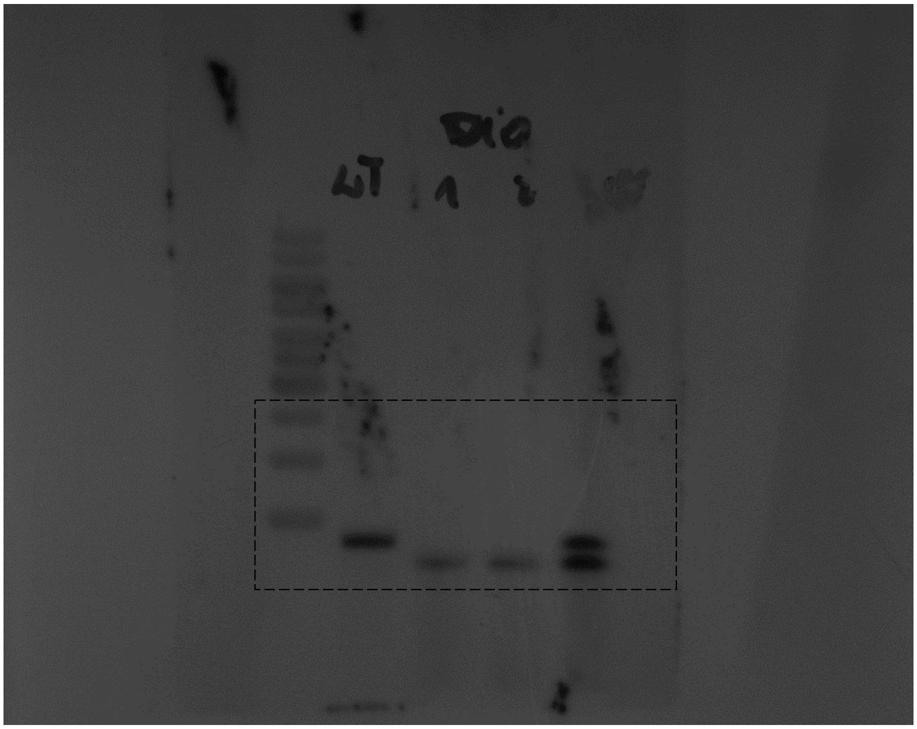

Western blot for *M. tuberculosis* strains probed with α-Rv3143 antibody

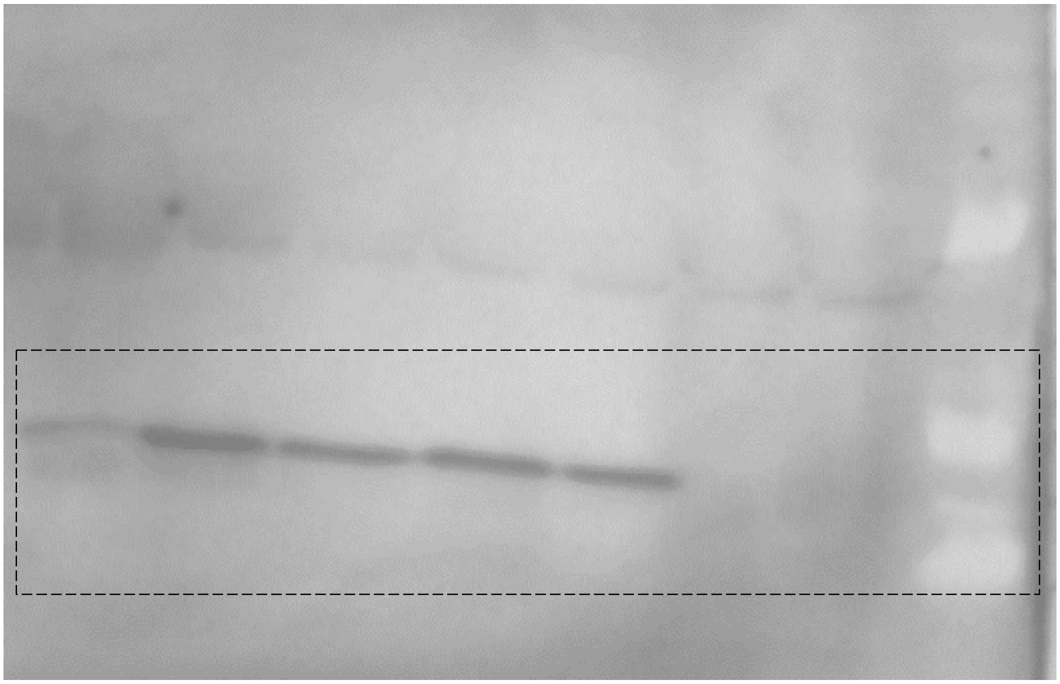

Western blot for *M. tuberculosis* strains probed with α-LigA antibody

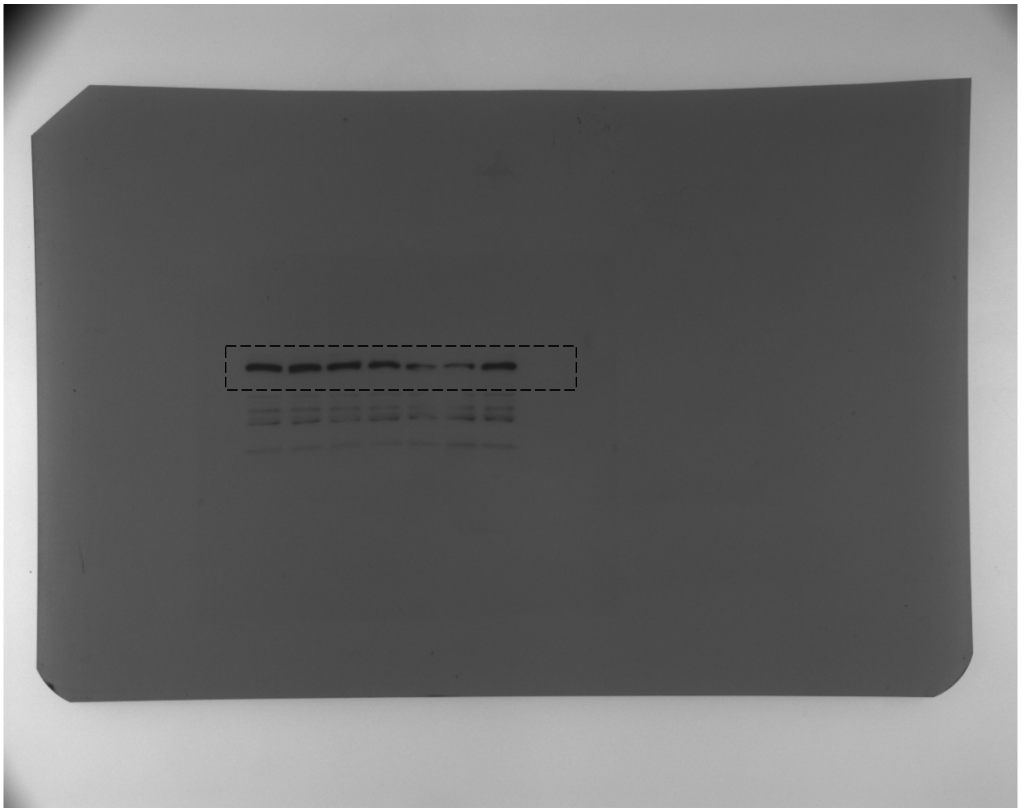

Southern blot confirmation of deletion of *M. smegmatis* mutant strains

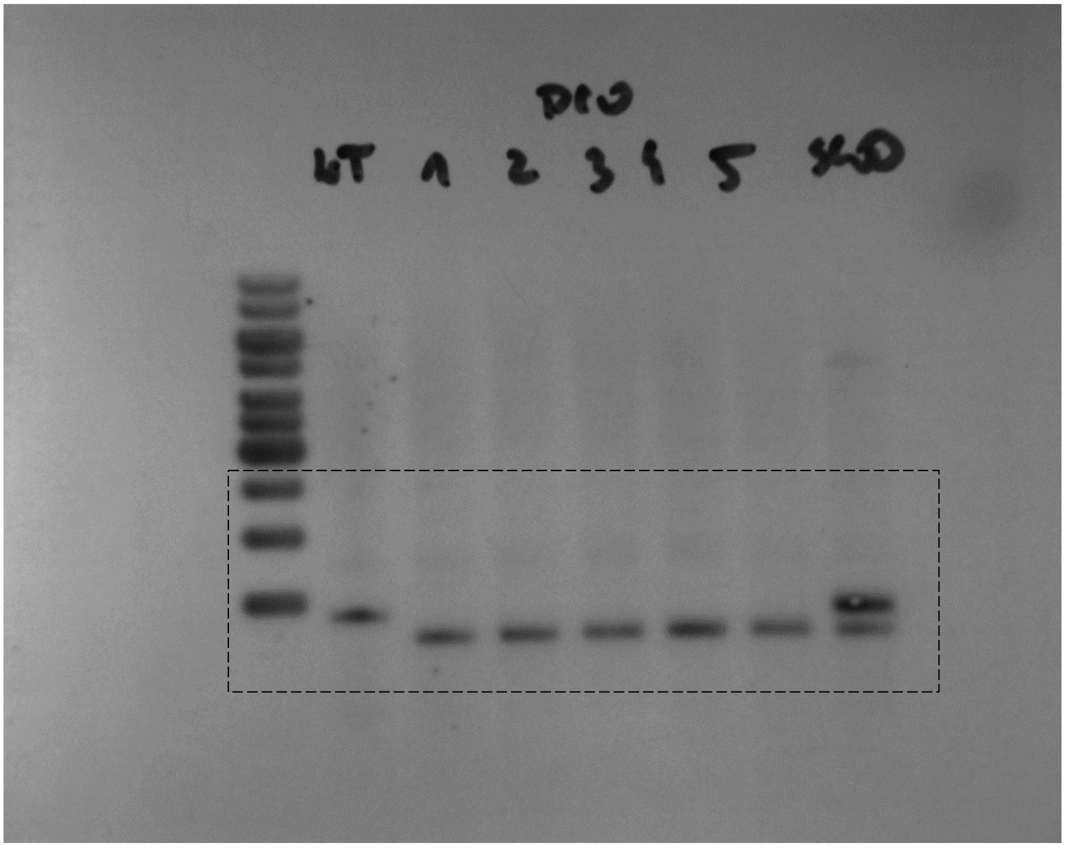

Figure S2.

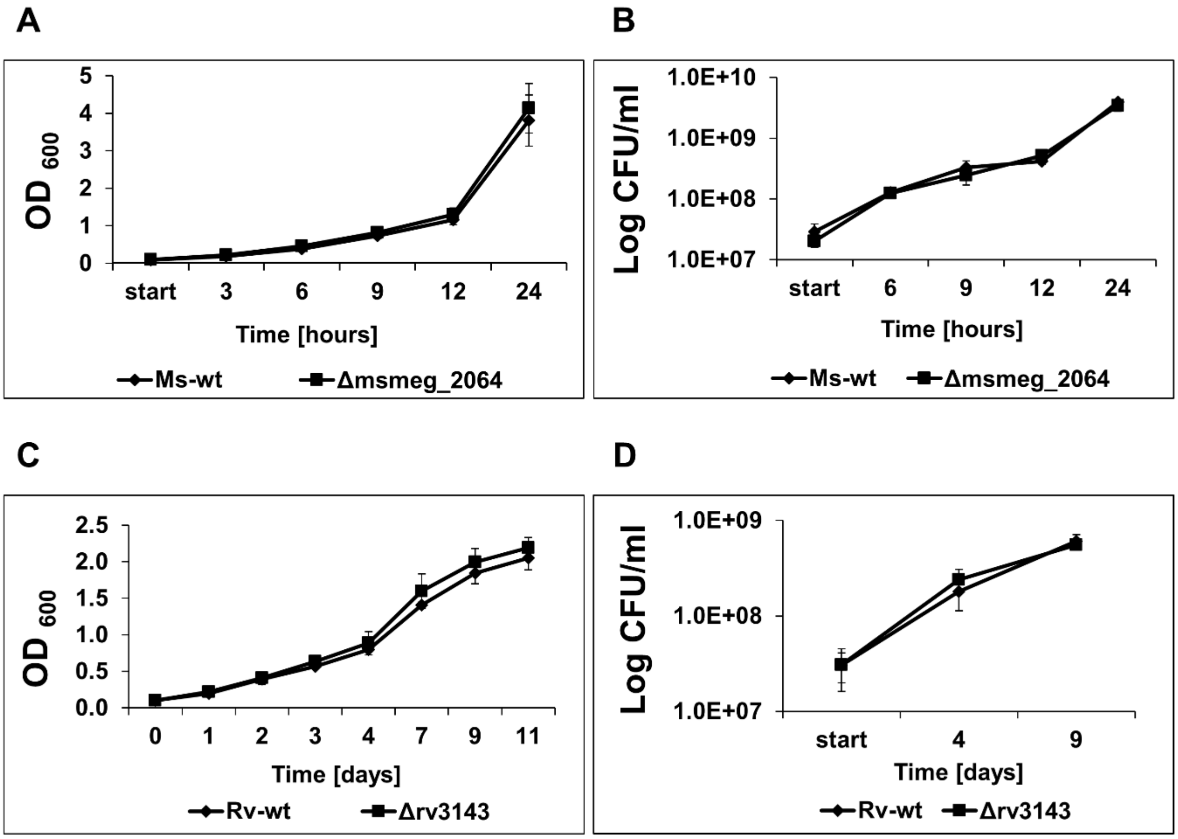

Figure S2. Phenotypic analyses of mutants defective in the synthesis of MSMEG_2064/Rv3143 response regulators in *M. smegmatis* (A, B) and *M. tuberculosis* (C, D). The mutant and wild-type strains were grown in 7H9 Middlebrook rich medium. The growth of strains was evaluated by measuring the OD_{600} at the indicated time points (A, C), and the numbers of viable cells were determined as bacterial colony forming units CFU/mL on 7H10/OADC plates (B, D). Means ± standard deviations are shown from three independent experiments.

Figure S3.

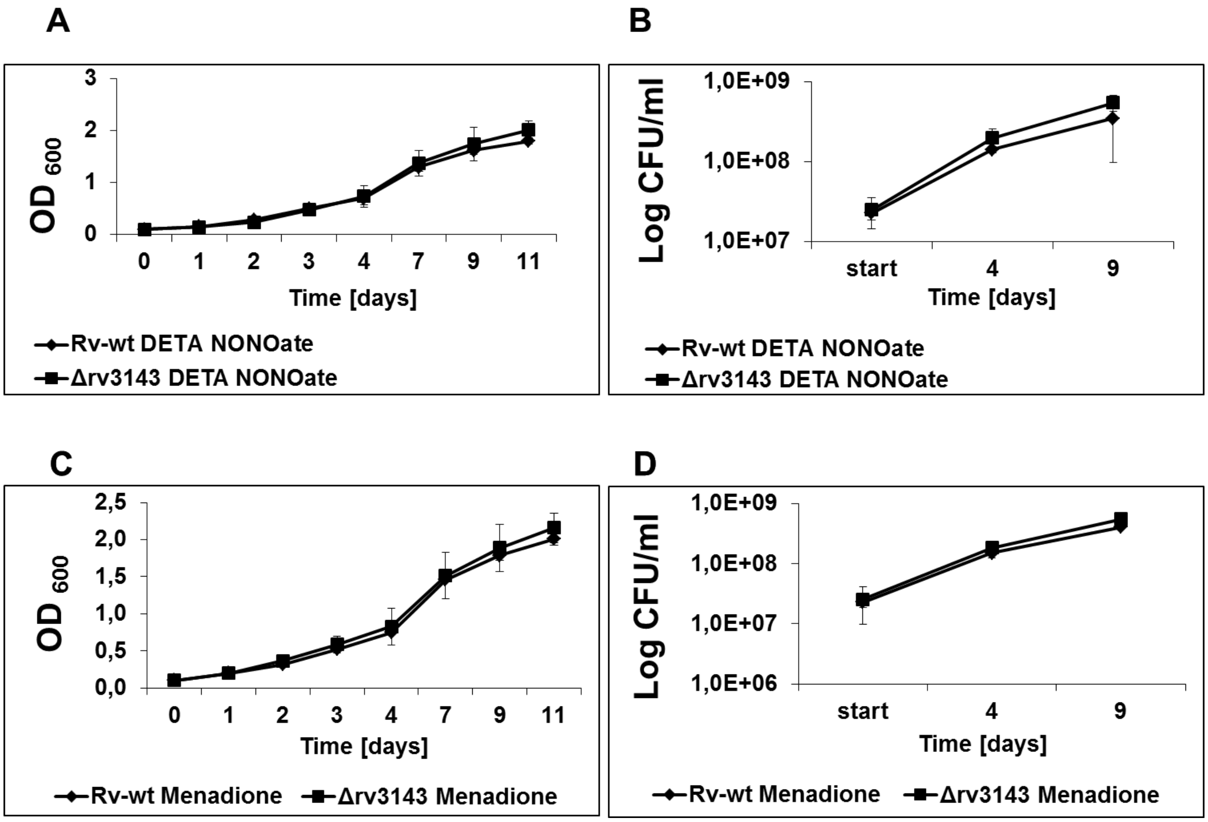

Figure S3. Phenotypic analyses of *M. tuberculosis* strains upon exposure to DETA NONOate (A, B) and menadione (C, D). The mutant Δ*rv3143* and wild-type Rv-wt strains were grown in 7H9 Middlebrook rich medium upon exposure to nitrosative (DETA NONOate 25 μM) and oxidative (menadione 10 μM) stress. The growth of strains was evaluated by measuring the OD_{600} at the indicated time points (A, C), and the numbers of viable cells were determined as bacterial colony forming units CFU/mL on 7H10/OADC plates (B, D). Means ± standard deviations are shown from three independent experiments.

Figure S4.

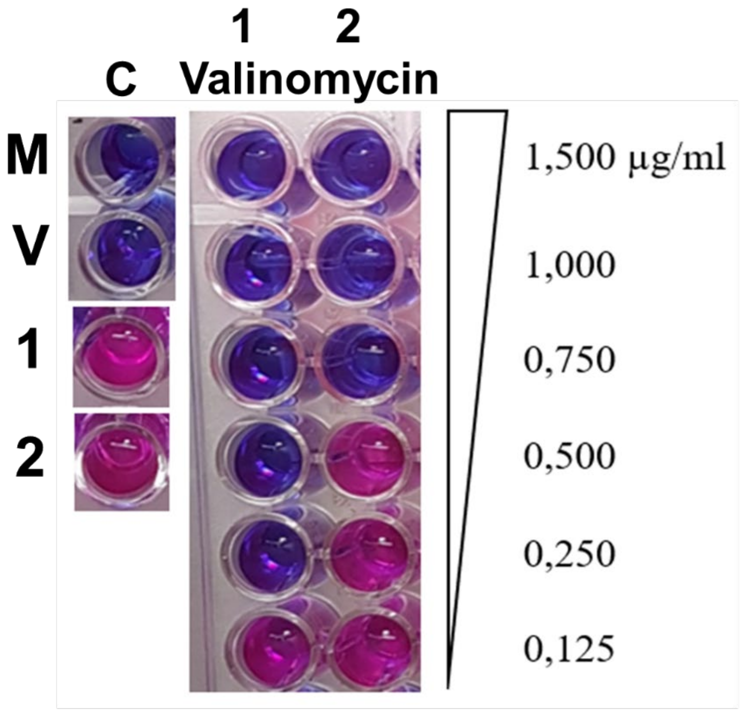

Figure S4. Evaluation of the susceptibility of *M. tuberculosis* wild type strain (1) and Δ*rv3143* mutant strain (2) to the tested concentrations of valinomycin. The experiments were performed in three biological repeats with the same results obtained. The figure shows the representative photo taken during a single experiment. The controls (C) performed in this experiment were wells filled with 7H9 medium (M), valinomycin (V) and strains without valinomycin.

Figure S5.

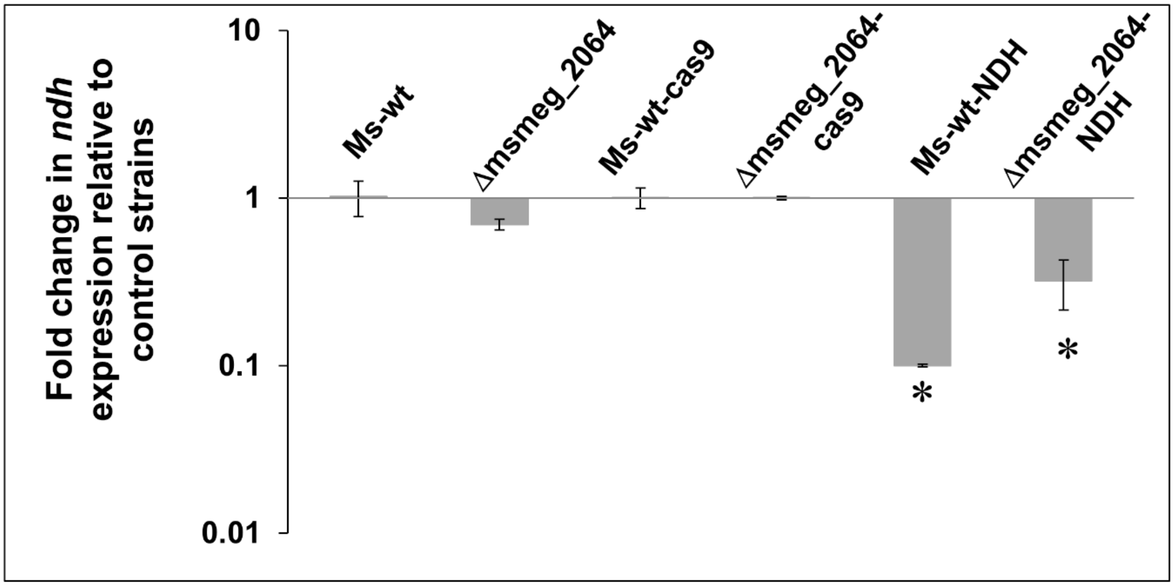

Figure S5. Expression profile of the *ndh* gene in *M. smegmatis* strains expressing decreased levels of the *ndh* gene. Mutant strains *ndh*$^{CRISPRi/dCas9}$ (Ms-wt-NDH), Δ*msmeg_2064-ndh*$^{CRISPRi/dCas9}$ (Δ*msmeg_2064*-NDH) and control strains MC^{2}155 (Ms-wt), CRISPRi/dCas9 (Ms-wt-cas9), Δ*msmeg_2064*-CRISPRi/dCas9 (Δ*msmeg_2064*-cas9) were grown in 7H9/AD broth for 16 hours at 37 °C with the addition of anhydrotetracycline (100 ng/µL) to deplete *ndh*. Transcript levels were determined using qRT-PCR and SYBR green chemistry. The expression levels of *ndh* were normalized to the *mysA* housekeeping gene and compared to the control strain. Statistical significance was determined using Student's t-test (∗ p <0,001).

Figure S6.

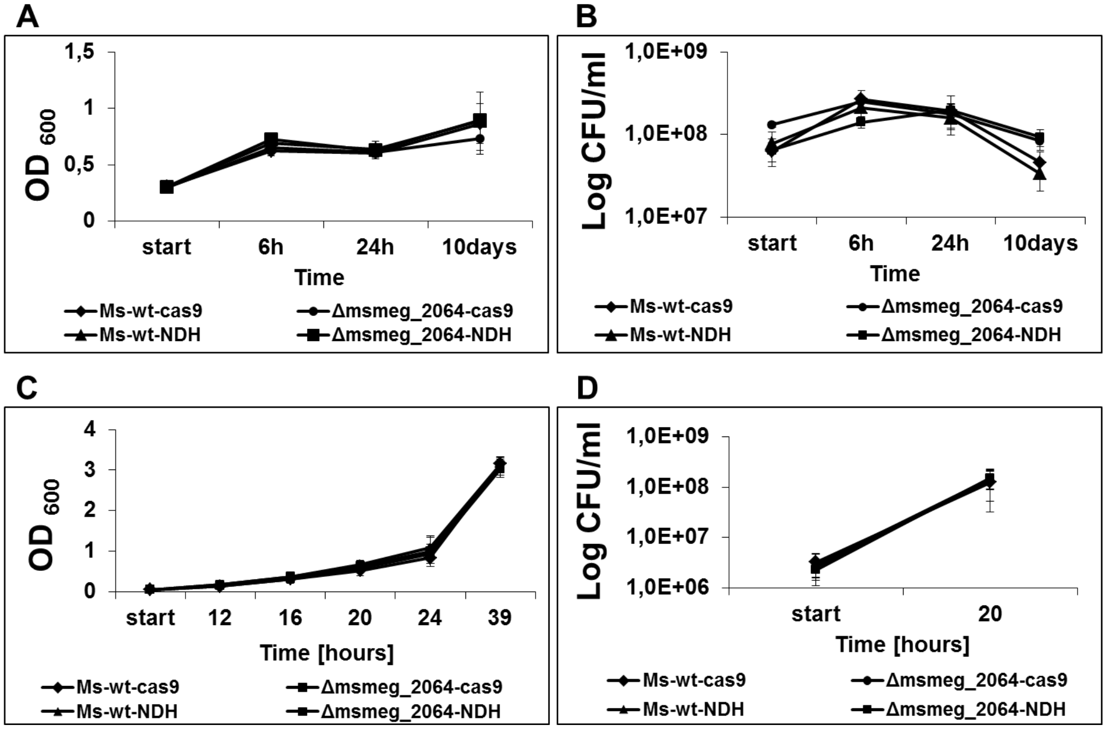

Figure S6. Kinetics of growth and viability of *M. smegmatis* strains expressing the functional *ndh* gene under hypoxia (A, B) and reaeration (C, D) conditions. The mutant *ndh*$^{CRISPRi/dCas9}$ (Ms-wt-NDH), Δ*msmeg_2064-ndh*$^{CRISPRi/dCas9}$ (Δ*msmeg_2064*-NDH) and the control strains CRISPRi/dCas9 (Ms-wt-cas9), Δ*msmeg_2064*-CRISPRi/dCas9 (Δ*msmeg_2064*-cas9*)* were grown in 7H9 Middlebrook broth supplemented with AD. The growth of strains was evaluated by measuring the OD_{600} at the indicated time points (A, C), and the numbers of viable cells were determined as bacterial colony forming units CFU/mL on 7H10/OADC plates (B, D). Means ± standard deviations are shown from three independent experiments.

Figure S7.

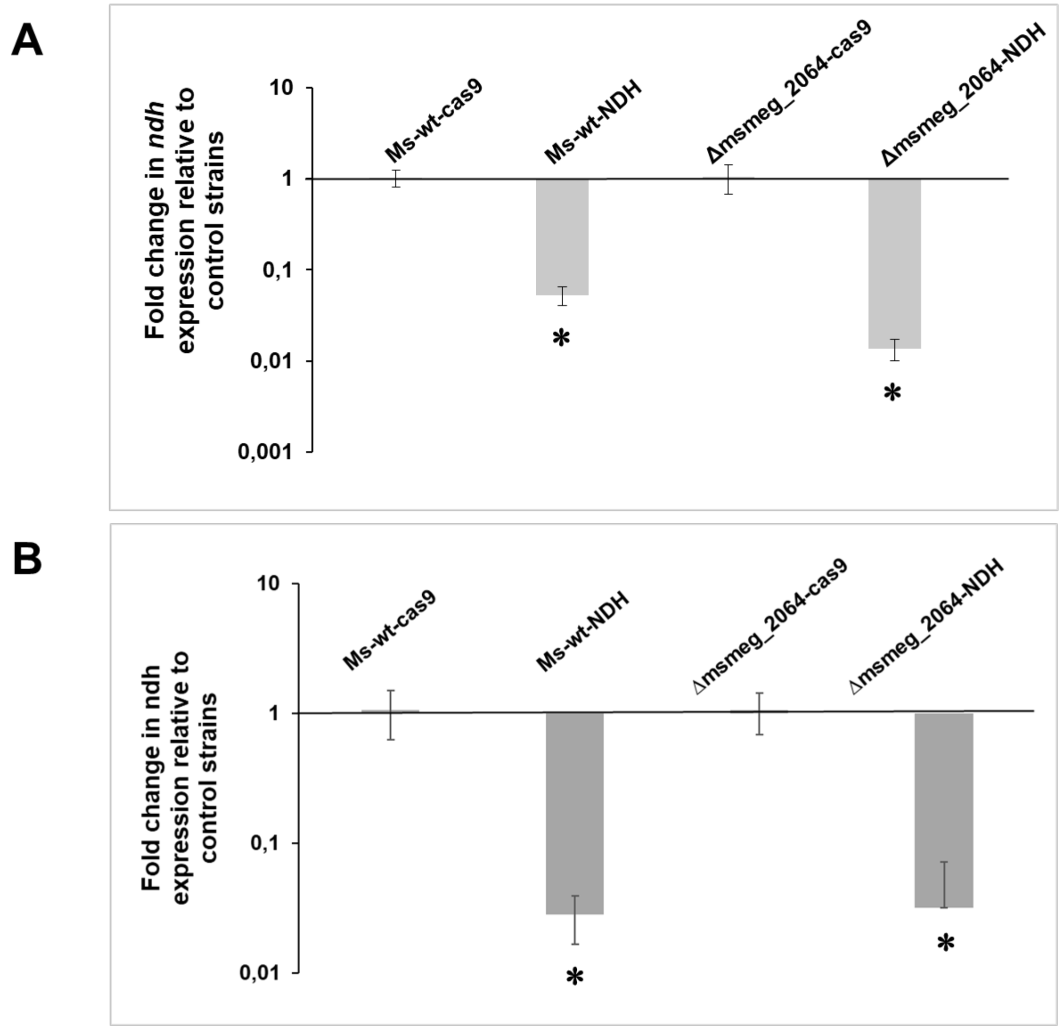

Figure S7. Expression profile of *ndh* gene in *M. smegmatis* strains under hypoxia for 24 h (A) and 10 days (B). The *M. smegmatis* wild-type and Δ*msmeg_2064* strains expressing "empty" CRISPRi/dCas9 vector and with inducible depletion of NDH ($ndh^{CRISPRi/dCas9}$) were grown in 7H9/AD medium with addition of aTc in order to silence the *ndh* gene. Transcript levels were determined using qRT-PCR and SYBR green chemistry. The expression levels of *ndh* were normalized to the *mysA* housekeeping gene

and compared to the control strain. Statistical significance was determined using Student's t-test (A) (∗p=0,001), (B) (∗p=0,015).

Original blots for Figure 2:

2B

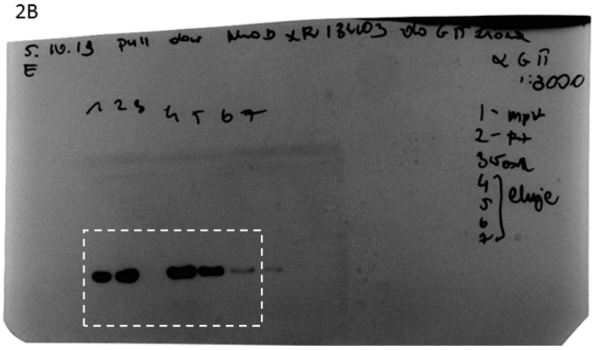

2A

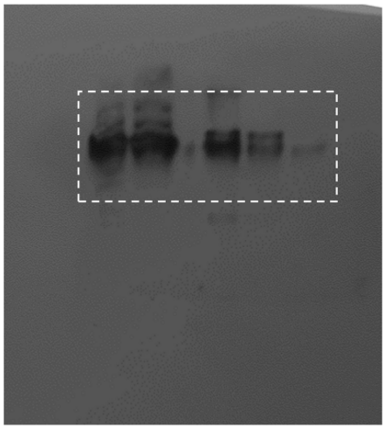

2E

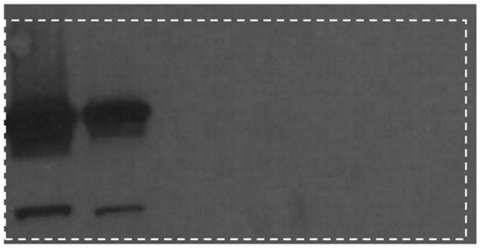

2D

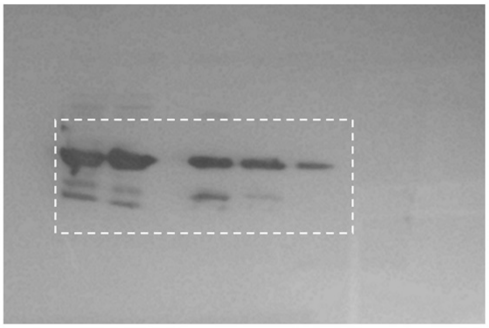

2C

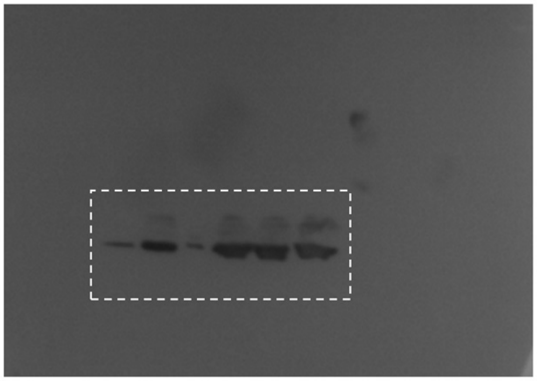

2F

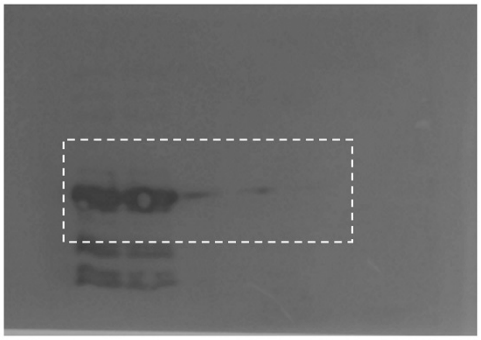

Additional control for Figure 2:

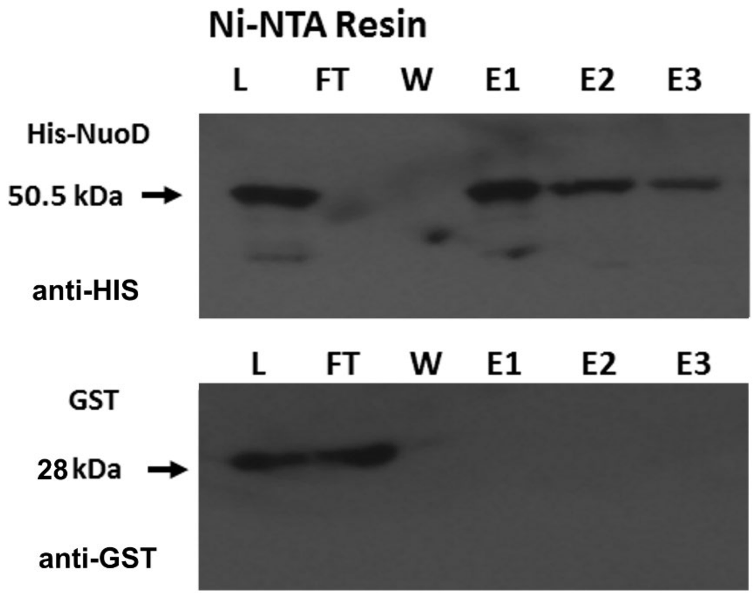

GST protein does not bind to NuoD and Ni-NTA resin. Immunoblots of His-NuoD pulled down with GST on Ni-NTA resin (A,B). Bound proteins were eluted with imidazole, resolved on SDS-PAGE gels, transferred to PVDF membranes and probed with α-His and α-GST antibodies. L-Load; FT- Flow through; W-Wash, E-Elution are shown.

Original blots of additional control for Figure 2:

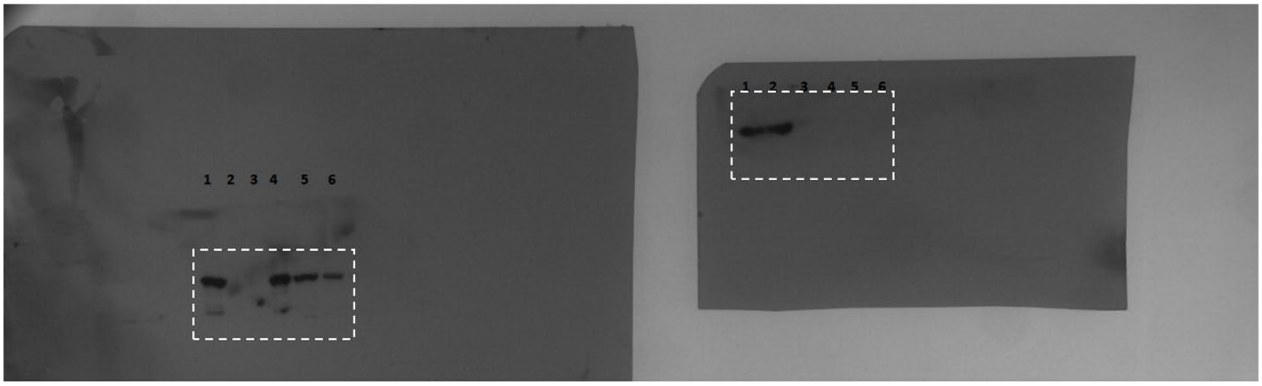

References:

1. Parish T, Stoker NG. 2000. Use of a flexible cassette method to generate a double unmarked Mycobacterium tuberculosis tlyA plcABC mutant by gene replacement. Microbiology (Reading, England) 146 (Pt 8:1969–1975.

2. Płociński P, Laubitz D, Cysewski D, Stoduś K, Kowalska K, Dziembowski A. 2014. Identification of protein partners in mycobacteria using a single-step affinity purification method. PloS one 9:e91380.

3. Williams KJ, Joyce G, Robertson BD. 2010. Improved mycobacterial tetracycline inducible vectors. Plasmid 64:69–73.

4. Rock JM, Hopkins FF, Chavez A, Diallo M, Chase MR, Gerrick ER, Pritchard JR, Church GM, Rubin EJ, Sassetti CM, Schnappinger D, Fortune SM. 2017. Programmable transcriptional repression in mycobacteria using an orthogonal CRISPR interference platform. Nature Microbiology 2:16274.
